# Assessment of the appropriateness of stress ulcer prophylaxis practice among hospitalized patients across Lebanese hospitals: A multicenter prospective study

**DOI:** 10.1371/journal.pone.0361969

**Published:** 2026-11-06

**Authors:** Khouloud Housary, Carole Dib, Rania Itani

**Affiliations:** 1 Pharmacy Practice Department, Faculty of Pharmacy, Beirut Arab University, Beirut, Lebanon; 2 Pharmacy Department, Lebanese Hospital Geitaoui University Medical Center, Beirut, Lebanon; Dana-Farber Cancer Institute, Harvard Medical School and THE Broad Institute of MIT and Harvard, LEBANON

## Abstract

**Background:**

Stress ulcer prophylaxis (SUP) is recommended for critically ill patients at high risk of stress-related gastrointestinal bleeding. Despite established guideline recommendations, inappropriate use of acid suppressant medications (ASMs), particularly proton pump inhibitors (PPIs), remains common worldwide and may expose patients to unnecessary adverse effects and increased healthcare costs. This study aimed to assess the appropriateness of SUP prescribing practices among hospitalized patients across Lebanese hospitals, focusing on indication, choice of pharmacologic agent, dosing regimen, route of administration, duration of therapy, discharge prescribing, and factors associated with inappropriate SUP practice.

**Methods:**

A multicenter prospective study was conducted between April 20 and May 22, 2026, across ten tertiary-care hospitals in different regions of Lebanon. Adult patients (≥18 years) admitted to Internal Medicine Unit, Intensive Care Unit (ICU), or Coronary Care Unit wards were assessed for the appropriateness of SUP, regardless of whether ASMs were prescribed. The appropriateness of SUP practice was evaluated according to the Society of Critical Care Medicine and American Society of Health-System Pharmacists (SCCM/ASHP) 2024 and ASHP 1999 guidelines. Multivariable logistic regression was performed to identify factors associated with inappropriate SUP practice.

**Results:**

A total of 552 patients were enrolled, with a mean age of 64.3 ± 18.5 years, and 58.7% were male. According to the SCCM/ASHP 2024 and ASHP 1999 guidelines, 90.8% and 77.2% of patients, respectively, were not eligible for SUP. Omeprazole was the most frequently prescribed agent (75.4%), and PPIs were used in all SUP prescriptions. Among patients admitted to ICU, 11.2% continued receiving ASMs after the SUP indication had resolved or following ICU transfer without a persisting indication. Overall, SUP practice was inappropriate in 77.9% of patients. Multivariable analysis demonstrated that anticoagulant therapy was associated with higher odds of inappropriate SUP practice (AOR = 2.78, 95% CI = 1.35–5.74, P = 0.006).

**Conclusions:**

Inappropriate SUP practice is highly prevalent among hospitalized patients in Lebanon, largely due to prescribing without guideline-based indications. Inappropriate continuation after resolution of the SUP indication or ICU transfer was also observed. Strengthening adherence to evidence-based recommendations through stewardship programs, prescribing audits, standardized protocols, and updated national guidance may help optimize SUP use and improve patient care.

## 1. Background

Stress-related mucosal disease (SRMD) includes a range of gastric mucosal abnormalities, from superficial stress-induced lesions to deeper, localized stress ulcers [1]. These lesions are most commonly seen in critically ill patients in intensive care units (ICUs) and are primarily caused by mucosal ischemia, as the gastric mucosa is highly susceptible to hemodynamic alterations and inflammatory mediators during critical illness [2]. SRMD is a well-recognized complication in this population and can lead to clinically significant gastrointestinal (GI) bleeding, thereby increasing both morbidity and mortality risk [3]. Proton pump inhibitors (PPIs) and histamine-2 receptor antagonists (H2RAs) represent effective pharmacological options for the prophylaxis of stress-related gastric lesions [2]. Both classes of drugs have been shown to reduce the risk of clinically significant gastrointestinal bleeding and are generally well tolerated [2].

The global prevalence of inappropriate stress ulcer prophylaxis (SUP), particularly involving PPI prescribing, remains notably high, with reported estimates ranging from 40% to 80% [4]. Similarly, a multicenter study conducted in Lebanon in 2011 across 16 hospitals demonstrated that approximately 67% of patients receiving SUP lacked a documented clinical indication [5]. In line with these findings, the ASHP 1999 therapeutic guidelines recommend limiting SUP to critically ill patients with clearly defined risk factors specifically those with one major criterion (mechanical ventilation for >48 hours or coagulopathy) or two or more minor criteria, such as sepsis, prolonged ICU stay, high-dose corticosteroid therapy, or severe trauma [6] Despite these well-established criteria, inappropriate SUP use has remained prevalent. More recently, the Society of Critical Care Medicine and American Society of Health-System Pharmacists (SCCM/ASHP) 2024 updated recommendations advocate a more restrictive, evidence-based approach, emphasizing that SUP should be reserved for ICU patients with coagulopathy, shock, or chronic liver disease, with regular reassessment and prompt discontinuation once indications resolve, particularly upon ICU transfer or hospital discharge [7].

The irrational or inappropriate use of SUP, particularly in the absence of evidence-based indications, has been extensively reported and is associated with unnecessary adverse events, increased rates of nosocomial infections such as hospital-acquired pneumonia and *Clostridioides difficile* infection. Studies have shown that inappropriate SUP, particularly with PPIs, increases the risk of *C. difficile* infection, with reported incidences ranging from 1.8% to 6.7% in ICU patients and a 38–40% higher relative risk compared with H2Ras [8,9], and higher healthcare costs, without demonstrable improvement in clinical outcomes [10] Notably, pharmacist-led interventions have been shown to significantly reduce both misuse and costs, from $26.75 and $2,433 to $7.14 and $239.80 per 100 patient-days (P < 0.001) [10].

Despite well-established clinical guidelines for SUP, inappropriate SUP prescribing remains highly prevalent both globally and at a national level. Although pharmacist-led interventions have demonstrated effectiveness in reducing inappropriate SUP use and associated expenditures, limited national studies have systematically evaluated the appropriateness of SUP prescribing across multiple Lebanese hospitals, including adherence to indications, choice of pharmacologic agent, dosing regimen, and prophylaxis duration. Consequently, there is a critical need to assess current SUP practices to identify gaps in guideline implementation and inform targeted strategies, including pharmacist-led interventions and policy development, to optimize patient safety and healthcare resource utilization.

The overall aim of the study was to assess the appropriateness of SUP practices among inpatients across Lebanese hospitals, focusing on the appropriateness of indications, choice of pharmacologic agent, dosing regimen, and duration of therapy according to established clinical guidelines.

## 2. Methods

### 2.1. Study design and population

This multicenter, prospective, observational, cross-sectional study was conducted across ten hospitals in different regions of Lebanon. The study was conducted from 20 April 2026 to 22 May 2026. The study population included hospitalized adult patients (aged 18 years and above) admitted to the Internal Medicine (IM), Intensive Care Unit (ICU), and Coronary Care Unit (CCU) wards. All adults admitted to these units during the study period were assessed for the appropriateness of SUP practice, regardless of whether ASMs were prescribed. This enabled the assessment of both appropriate and potentially inappropriate use, as well as potential underuse of SUP.

Patients were excluded if they had a history of active GI ulceration or GI bleeding within the previous year, presented with active gastrointestinal bleeding or an active peptic, gastric, or duodenal ulcer at admission or during hospitalization, used non-steroidal anti-inflammatory drugs (NSAIDs) before or during hospitalization, or had received ASMs prior to hospital admission.

### 2.2. Study setting and pharmacy practice experience V (PPE V) course overview

The study was conducted across ten large tertiary-care teaching hospitals in Lebanon, including nine private hospitals and one public hospital. The participating hospitals were geographically distributed across three major urban centers: seven hospitals were located in Beirut, the capital of Lebanon; two were located in Saida, the capital of South Lebanon; and one was located in Tripoli, the capital of North Lebanon. The participating hospitals were large healthcare institutions providing tertiary-level and specialized medical services to diverse patient populations. The private hospitals served patients from varied socioeconomic backgrounds, whereas the public hospital primarily served individuals from low- and middle-income groups. Collectively, these institutions provided care across multiple medical specialties and served diverse patient populations, providing a broad study setting for assessing the SUP practice. To maintain institutional confidentiality, individual hospitals are not identified.

The prescribing practices of SUP were observed, monitored, and documented by senior pharmacy students from the Faculty of Pharmacy at Beirut Arab University (BAU) during their clinical rotations at affiliated hospitals as part of the Pharmacy Practice Experience V (PPE V) course requirement. The PPE V course is an advanced experiential course within the pharmacy curriculum. This course is introduced during the fifth year of the Bachelor of Pharmacy program and consists of four consecutive months of clinical rotations in tertiary-care hospitals. The course enabled students to participate in the responsibilities of a clinical pharmacist, providing practical experience that facilitated their transition from students into competent, practice-ready pharmacists. During the course, students delivered optimal patient care, drug information, health promotion and disease prevention services, and medication therapy management under the close supervision of their preceptors.

The PPE V course consisted of four clinical rotations, each lasting one month. The rotations involved assigning students to clinical wards, where they were guided and supervised by skilled faculty and site preceptors, as well as medical coordinators, throughout their internship. These preceptors also oversaw and supported the site-related activities performed by students on the medical wards.

### 2.3. Data collection form development

Data were collected using a structured data collection form developed specifically for this study following an extensive literature review, including relevant studies and applicable clinical guidelines [1,5,7,10–16].

The data collection form consisted of four sections. The full data collection form is provided in S1 Appendix. **Section one** retrieved patients’ sociodemographic and hospitalization characteristics, including age, sex, hospital type (teaching or non-teaching), admitting unit (IM, ICU, or CCU), length of hospital stay, and prescriber level and specialty.

**Section two** focused on collecting clinical variables and common risk factors associated with stress-related gastrointestinal bleeding. These included admission to the ICU, ICU length of stay, coagulopathy parameters, invasive mechanical ventilation for more than 48 hours, history of gastrointestinal ulceration or bleeding, Glasgow Coma Scale score, multiple trauma, traumatic brain injury, spinal cord injury, partial hepatectomy, chronic liver disease, thermal injury involving more than 35% of the body surface area, hepatic or renal transplantation, shock, high-dose glucocorticoid therapy, and occult or overt GI bleeding lasting six days or longer.

**Section three** documented and assessed the appropriateness of SUP practices by determining whether patients met the eligibility criteria outlined in the American Society of Health-System Pharmacists (ASHP) guidelines (1999 and 2024) [6,7]. **Appropriateness of SUP use based on clinical indication** was defined as the prescription of SUP to a patient with an eligible clinical indication according to either the 1999 ASHP or 2024 SCCM/ASHP guidelines, or the absence of SUP prescription when no indication was identified according to either guideline. Conversely, SUP use was considered inappropriate when prophylaxis was prescribed despite the absence of an indication according to both guidelines (**overutilization**) or when prophylaxis was not prescribed despite the presence of an indication according to either guideline (**underutilization**) [6,7].

**Section four** evaluated ASM prescribing patterns and appropriateness among patients who received ASMs during hospitalization. The assessment included the type of ASM prescribed, including proton pump inhibitors, histamine-2 receptor antagonists, antacids, gastrointestinal mucosal protectants (sucralfate), and prostaglandin analogs (misoprostol), as well as the prescribed dose, route of administration, and duration of prophylaxis. The appropriateness of the prescribed agent, dose, and duration was assessed according to guideline-defined criteria. The assessment also documented continuation of ASM at hospital discharge. **Overall SUP prescribing appropriateness** was determined by considering indication, selected agent, dose, and duration, with overall practice classified as appropriate only when all assessed components met the predefined appropriateness criteria.

### 2.4. Data collection form revision and pilot testing

To optimize the data collection tool, the form underwent assessment for face and content validity by an expert panel comprising three clinical and hospital pharmacy specialists. Their feedback was used to enhance the form, improve clarity, and ensure alignment with routine practices across various clinical settings.

The finalized version was pilot-tested by a group of senior pharmacy students during their inpatient clinical rotations to evaluate usability, completeness, and feasibility in terms of time requirements. Based on student feedback and observed documentation practices, minor revisions were implemented. Data generated during the pilot phase were excluded from the final analysis.

### 2.5. Data collectors’ training

Data collectors, consisting of senior pharmacy students assigned to different affiliated hospitals, received structured training delivered by the principal investigators (PIs) through a series of training sessions and workshops. These sessions familiarized students with SUP clinical guidelines, risk factor assessment, and standardized data collection procedures. Practical exercises and case-based discussions were incorporated to enhance students’ ability to accurately classify SUP use as appropriate or inappropriate.

The training ensured competency in reviewing patients’ medical records for the evaluation of SUP practices. Emphasis was placed on identifying indications for SUP and assessing the appropriateness of selected agents, dosing regimens, routes of administration, and duration of therapy according to established clinical guidelines [6,7].

During the data collection phase, students were supervised by their assigned faculty preceptors as part of their routine hospital activities to ensure accurate extraction and documentation of relevant data using the standardized data collection form. All completed forms were reviewed and validated by faculty preceptors before being compiled and submitted to the PIs for final analysis.

### 2.6. Ethical considerations

This study adhered to the ethical standards outlined in the World Medical Association’s Declaration of Helsinki [17]. Ethical approval was obtained from the Institutional Review Board (IRB) at Beirut Arab University on April 16, 2026; no approval code was issued. The IRB confirmed that the study was approved in accordance with institutional regulations and that data collection could proceed. As the study involved prospective review of medical records without direct patient contact, no patient identifiers were collected, and all data were handled confidentially. Access was restricted to the principal investigators, with secure storage of electronic and physical records. Only anonymized datasets were shared with participating institutions. A waiver of informed consent was granted by the IRB due to the observational nature of the study and absence of direct patient interaction.

### 2.7. Data preparation and analysis

Upon completion of data collection, all submitted forms underwent quality review to assess completeness, readability, internal consistency, and clarity. Each recorded case was verified by the corresponding faculty preceptor. Forms containing missing information, unclear entries, or inconsistent responses were excluded from the analysis. Consequently, no missing data were present in the final dataset used for the statistical analyses.

Data analysis was performed using the IBM Statistical Package for Social Sciences (SPSS^®^) version 26.0 (IBM Corp., Armonk, NY, USA). Descriptive statistics were reported as frequencies and percentages for categorical variables and as means with standard deviations for continuous variables. Univariate analyses were performed to examine the association between potential predictors and inappropriate SUP practice using Pearson’s chi-square (χ^2^) test for categorical variables, and independent t-test for continuous variables. Variables with a P-value < 0.25 in univariate testing were considered for inclusion in the multivariable logistic regression model. The variables entered into the initial multivariable model were hospital affiliation status (including university-affiliated *vs*. non-university-affiliated hospital), clinician in charge, length of hospital stay, ICU stay (including ICU ≤ 1 week *vs* > 1 week), and receipt of anticoagulant therapy (yes *vs.* no).

Multicollinearity was evaluated using variance inflation factors (VIFs), with a cut-off value of 10 for exclusion. All VIFs were below 2.5, indicating no evidence of significant multicollinearity among predictors. Variables were further assessed for clinical relevance, with conceptually related factors reviewed for potential inclusion.

The final multivariable logistic regression model was constructed using a backward stepwise selection procedure, in which all candidate variables were initially entered into the model and subsequently removed in a stepwise manner according to their statistical contribution. Model fit was assessed using the Hosmer–Lemeshow goodness-of-fit test. The final model demonstrated adequate fit (χ^2^ = 4.574, df = 5, P = 0.470). Adjusted odds ratios (AORs), along with 95% confidence intervals (CIs) and P-values, were reported for variables retained in the final model. Statistical significance was set at P < 0.05.

## 3. Results

### 3.1. Participants’ sociodemographic and clinical characteristics

Table 1 describes the patients’ sociodemographic and clinical admission characteristics. A total of 552 patients were enrolled in the study. The mean age of the participants was 64.31 ± 18.49 years, with more than half being elderly patients aged ≥ 65 years (n = 310, 56.2%). Male patients constituted 58.7% of the study population (n = 324). In terms of hospital units, the majority of patients were treated in the IM unit (n = 292, 52.9%), followed by the ICU (n = 204, 37.0%) and the CCU (n = 56, 10.1%).

**Table 1:** Patients’ sociodemographic and clinical admission characteristics (N = 552).

Item	n (%)
**Sex**	
Male	324 (58.7)
Female	228 (41.3)
**Age (in years)**	
Mean ± SD = 64.31 ± 18.49 (range = 22–98)	
< 45	104 (18.8)
45–64	138 (25)
≥ 65	310 (56.2)
**Hospital Unit**	
Internal Medicine (IM)	292 (52.9)
Intensive Care Unit (ICU)	204 (37)
Cardiac Care Unit (CCU)	56 (10.1)
**Duration of Hospitalization (in days)**	
Mean ± SD = 6.05 ± 11.04 (range = 1–180)	

Regarding the responsible clinicians for patients’ cases, nearly half were residents (n = 268, 48.6%), followed by general physicians/hospitalists (n = 202, 36.6%), while specialists/attending physicians accounted for 13.8% (n = 76). Among specialist/attending physicians, Internal Medicine physicians were the most frequent specialists, accounting for (n = 31, 40.8%), followed by Gastroenterology specialists (n = 18, 23.7%) and Cardiology specialists (n = 13, 17.1%). While the less common responsible clinicians were Pulmonology (n = 7, 9.2%), Emergency (n = 3, 3.9%), Infectious Diseases (n = 2, 2.6%), Nephrology (n = 1, 1.3%), and Hematology/Oncology specialists (n = 1, 1.3%).

### 3.2. Clinical risk factors for stress ulcer development

Table 2 summarizes the clinical risk factors for stress ulcer development among the study participants (N = 552). ICU stay was the most commonly identified risk factor, reported in 37.1% (n = 205) of patients. Among ICU patients, nearly one-third had an ICU stay exceeding one week (n = 64, 31.2%). Antiplatelet therapy was administered to 31.3% (n = 173) of the patients, of whom most received a single antiplatelet agent (n = 120, 69.3%), whereas dual antiplatelet therapy was reported in 30.7% (n = 53). While anticoagulant use was identified in 30.8% (n = 170) of patients.

**Table 2:** Clinical risk factors for stress ulcer development among patients (N = 552).

Item	n (%)
ICU Stay	205 (37.1)
If the patient is in the ICU, his/her stay is for > 1 week	
No	141 (68.8)
Yes	64 (31.2)
Antiplatelet intake	173 (31.3)
Single antiplatelet therapy	120 (69.3)
Dual antiplatelet therapy	53 (30.7)
Anticoagulant intake	170 (30.8)
On mechanical ventilation for > 48hrs	83 (15)
Glasgow Coma score of ≤ 10, or inability to obey simple commands	52 (9.4)
Coagulopathy (i.e., platelet count of < 50,000 mm3, INR of ≥ 1.5, or PTT > 2 times thecontrol)	34 (6.2)
Shock	34 (6.2)
Hepatic failure (chronic liver disease)	29 (5.3)
NSAIDs intake > 3 months	29 (5.3)
Glucocorticoid therapy (>250 mg hydrocortisone or the equivalent)	27 (4.9)
Multiple trauma or traumatic brain injury (injury severity score of ≥ 16)	23 (4.2)
History of gastrointestinal ulceration or bleeding within 1 year of admission	5 (0.9)
Spinal cord injury	2 (0.4)
Partial hepatectomy	2 (0.4)
Occult or Overt GI bleeding lasting ≥ 6 days	2 (0.4)
Thermal injury to > 35% of body surface area	2 (0.4)
Hepatic or renal transplantation	0 (0)

A total of 15% of the patients were put on mechanical ventilation for more than 48 hours (n = 83), while 9.4% of patients had a Glasgow Coma Scale score ≤ 10 (n = 52). Coagulopathy and shock were each reported in 6.2% (n = 34) of patients. Hepatic failure or chronic liver disease and prolonged NSAID use for more than three months were both identified in 5.3% (n = 29) of patients. Glucocorticoid therapy equivalent to > 250 mg hydrocortisone was administered to 4.9% (n = 27) of patients, while multiple trauma or traumatic brain injury occurred in 4.2% (n = 23).

Less frequently reported risk factors included a history of gastrointestinal ulceration or bleeding within one year prior to admission (n = 5, 0.9%). Spinal cord injury, partial hepatectomy, occult or overt gastrointestinal bleeding lasting ≥ 6 days, and thermal injury involving >35% of body surface area was each documented in 0.4% (n = 2) of patients. No patients had a history of hepatic or renal transplantation.

### 3.3. Assessment of SUP indications according to ASHP guidelines

Based on the SCCM/ASHP Guidelines 2024, the majority of patients did not have an indication for SUP (n = 501, 90.8%), whereas only 9.2% (n = 51) met the criteria for SUP use. Similarly, according to the ASHP Guidelines 1999, most patients did not have an indication for SUP (n = 426, 77.2%), while 22.8% (n = 126) were considered eligible for SUP, and all eligible patients were critically ill in addition to at least one major or at least two or more minor risk factors for stress ulcer development.

### 3.4. Evaluation of the appropriateness of SUP use based on clinical indications

Fig 1 illustrates the appropriateness of SUP use based on clinical indications among the study participants (N = 552). Patients were classified as having an indication for SUP if they met the eligibility criteria of either the 1999 ASHP or the 2024 SCCM/ASHP guidelines, whereas those who did not meet the criteria of either guideline were classified as having no indication.

**Fig 1:**
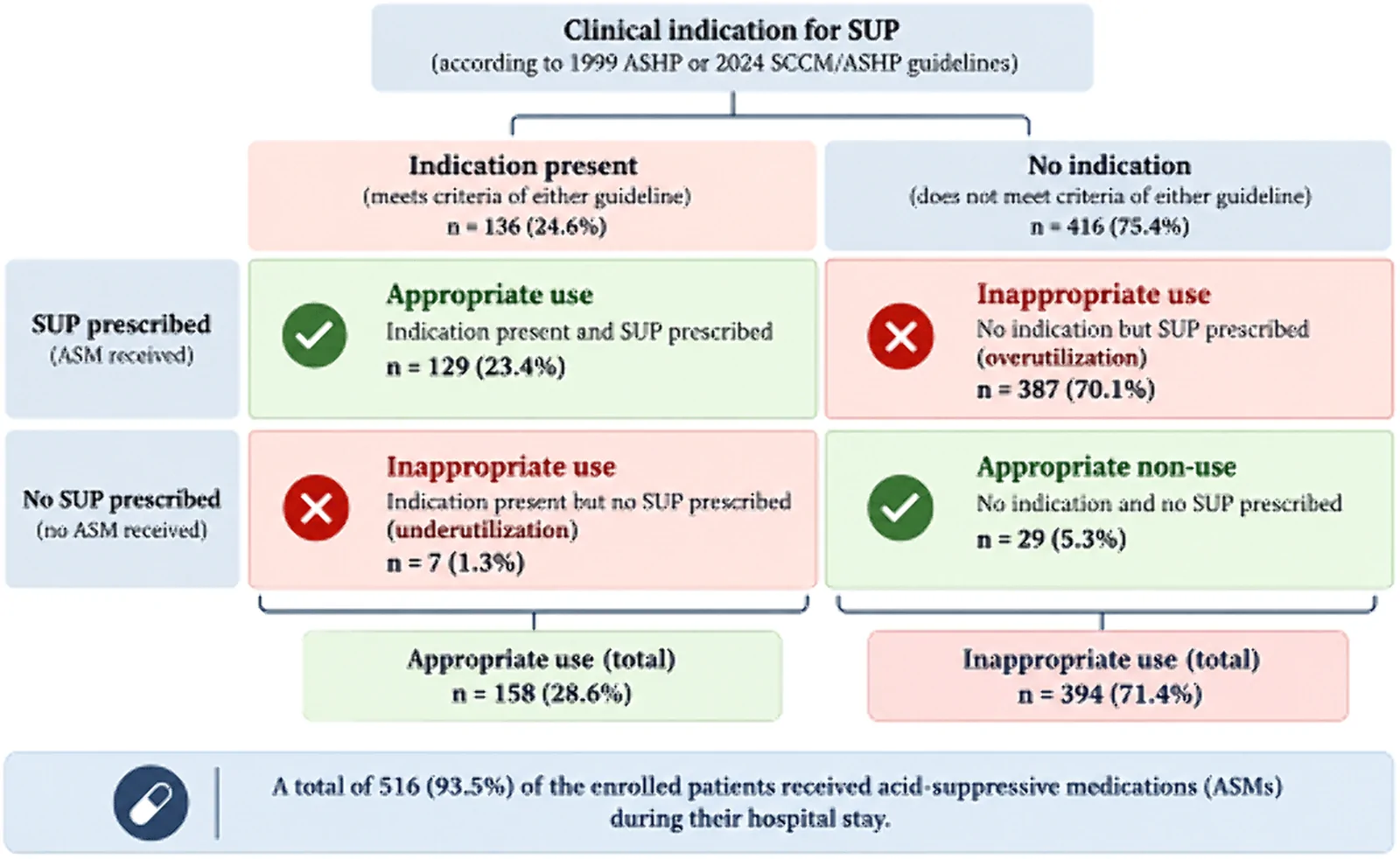
Appropriateness of SUP use based on clinical indications among study participants (N = 552). ASHP, American Society of Health-System Pharmacists; ASM, acid-suppressive medication; SCCM, Society of Critical Care Medicine; SUP, stress ulcer prophylaxis.

Overall, inappropriate SUP use was identified in 71.4% (n = 394) of patients. The majority of patients (70.1%, n = 387) received SUP despite the absence of a guideline-defined indication, representing overutilization. Conversely, 1.3% (n = 7) had an indication for SUP but did not receive prophylaxis, representing underutilization.

Appropriate SUP use was observed in 28.6% (n = 158) of patients. Specifically, 23.4% (n = 129) had a guideline-defined indication and appropriately received SUP, while 5.3% (n = 29) had no indication and appropriately did not receive prophylaxis.

Overall, 516 (93.5%) of the enrolled patients received acid-suppressive medications (ASMs) during their hospital stay.

### 3.5. Patterns of ASM use and prescribing appropriateness

Table 3 summarizes the patterns of ASM use, prescribing appropriateness, and discharge prescribing among patients who received ASMs during hospitalization (N = 516).

**Table 3:** Evaluation of acid suppressant medications (ASM) use (N = 516).

Item	n (%)
**Acid Suppressant Medications (ASM) Used Among Patients**	
Omeprazole	389 (75.4)
Esomeprazole	59 (11.4)
Pantoprazole	47 (9.1)
Rabeprazole	21 (4.1)
**Initiated Dose**	
20 mg	38 (7.4)
40 mg	478 (92.6)
**Dose Appropriateness**	
No	16 (3.1)
Yes	500 (96.9)
**Route of Administration**	
Oral	62 (12)
Parenteral (IV)	449 (87)
Enteral (gastrostomy/PEG tube/nasogastric)	5 (1)
**Duration of ASM (in days)**	
Mean ± SD = 6.05 ± 11.04 (range = 1–61)	
**ASM Duration Appropriateness**	
**Patients with ICU admission**	
Appropriate duration: discontinued when SUP indication resolved/ at ICU transfer when no longer indicated	175 (88.8)
Inappropriate duration: continued after indication resolved/ after ICU transfer without indication	22 (11.2)
**Patients without ICU admission (IM/CCU only)**	
Inappropriate from initiation, no guideline-defined SUP indication	319 (100)
Appropriate, Guideline-defined indication present	0 (0)
**Prescription of ASM at Discharge**	
No	475 (92.1)
Yes	41 (7.9)
**Evaluation of Overall SUP Practice (Indication, Dose, and Duration)**	
Appropriate	114 (22.1)
Inappropriate	402 (77.9)

Omeprazole was the most commonly prescribed ASM (n = 389, 75.4%), followed by esomeprazole (n = 59, 11.4%), pantoprazole (n = 47, 9.1%), and rabeprazole (n = 21, 4.1%). Most patients received a daily dose of 40 mg (n = 478, 92.6%), whereas 38 (7.4%) received 20 mg. The prescribed dose was considered appropriate in 500 (96.9%) patients and inappropriate in 16 (3.1%).

Regarding the route of administration, the intravenous/parenteral route was most frequently used (n = 449, 87.0%), followed by oral administration (n = 62, 12.0%) and enteral administration via gastrostomy, percutaneous endoscopic gastrostomy (PEG), or nasogastric tube (n = 5, 1.0%). The mean duration of ASM use was 6.05 ± 11.04 days, ranging from 1 to 61 days. Among the 197 patients admitted to the ICU, ASM duration was considered appropriate in 175 (88.8%), as therapy was discontinued when the SUP indication resolved or at ICU transfer when no longer indicated, whereas 22 (11.2%) had an inappropriate duration due to continuation after resolution of the indication or following ICU transfer without a persisting indication. Among the 319 patients who were not admitted to the ICU, all received ASM without a guideline-defined indication for SUP and were therefore considered to have inappropriate therapy from initiation rather than inappropriate duration.

At hospital discharge, 41 (7.9%) patients continued to receive an ASM. When the overall appropriateness of SUP management was evaluated based on indication, dose, and duration, 402 (77.9%) patients had at least one inappropriate component, whereas 114 (22.1%) met all predefined appropriateness criteria (Fig 2).

**Fig 2:**
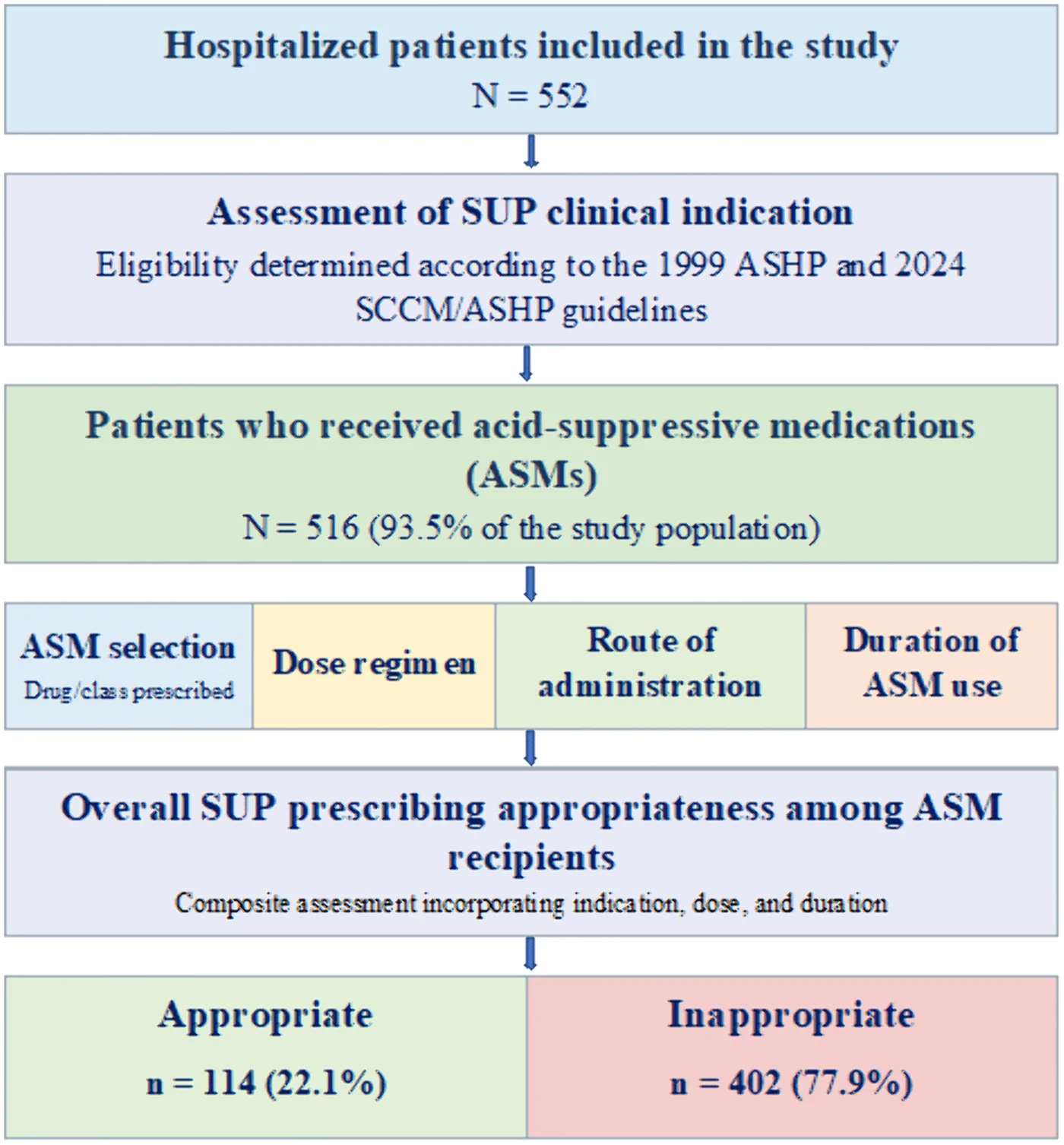
Study flowchart for assessment of SUP indication and ASM prescribing appropriateness. ASHP, American Society of Health-System Pharmacists; ASM, acid-suppressive medication; SCCM, Society of Critical Care Medicine; SUP, stress ulcer prophylaxis.

### 3.6. Predictors of overall inappropriate acid suppressant medication use in stress ulcer prophylaxis practice among study participants (N = 516)

Table 4 presents the univariate analysis of predictors associated with the overall inappropriate use of ASMs for SUP among the study patients (N = 516). A total of 552 patients were initially screened for SUP eligibility, of whom 36 were excluded following risk-stratification assessment because they either had no indication for prophylaxis or had an indication but did not receive ASM. Thus, the final study cohort comprised 516 patients who received ASMs and were included in the analysis. Hospital affiliation status was significantly associated with inappropriate SUP practice, where patients admitted to university-affiliated hospitals were more likely to experience inappropriate SUP practice compared to those admitted to non-university-affiliated hospitals (UOR = 1.90, 95% CI = 1.25–2.89, P = 0.003).

**Table 4:** Univariate analysis of predictors associated with overall inappropriate use of acid suppressant medications for stress ulcer prophylaxis practice (N = 516).

Item	n (%)^a^	SUP Practice		UOR (95% CI)	*P* ^*c*^
		Appropriate n = 114 (%)^b^	Inappropriate n = 402 (%)^b^		
**Hospital Affiliation Status** (reference: non-university affiliated)^**†**^					
Non-University Affiliated	217 (42.1)	62 (28.6)	155 (71.4)	–	0.003^d^
University Affiliated	299 (57.9)	52 (17.5)	247 (82.6)	1.90 (1.25-2.89)	
**Clinician in charge** (reference: General physician)^**†**^					
Intern	5 (1)	0 (0)	5 (100)	–	<0.001^d^
Resident	254 (49.2)	41 (16.1)	213 (83.9)	3.11 (1.99-4.86)	
General Physician	187 (36.2)	70 (37.4)	117 (62.6)	–	
Internal Medicine	31 (6)	0 (0)	31 (100)	–	
Gastroenterology	16 (3.1)	0 (0)	16 (100)	–	
Cardiology	12 (2.3)	2 (16.7)	10 (83.3)	2.99 (0.64-14.1)	
Pulmonology	6 (1.2)	1 (16.7)	5 (83.3)	2.99 (0.34-26.1)	
Nephrology	1 (0.2)	0 (0)	1 (100)	–	
Infectious Disease	2 (0.4)	0 (0)	2 (100)	–	
Hematology/Oncology	1 (0.2)	0 (0)	1 (100)	–	
Emergency Medicine	1 (0.2)	0 (0)	1 (100)	–	
**Length of Hospital Stay** ^**†**^					
Mean ± SD	6.19 ± 11.40	11.16 ± 21.91	4.78 ± 4.75	–	0.003^d,e^
**Age Group** (reference: < 45 years)					
< 45 years	96 (18.6)	23 (24)	73(76)	–	0.23
45–64 years	131 (25.4)	22 (16.8)	109 (83.2)	1.56 (0.81-3.01)	
≥ 65 years	289 (56)	69 (23.9)	220 (76.1)	1.00 (0.58-1.73)	
**Gender** (reference: Male)					
Male	301 (58.3)	68 (22.6)	233 (77.4)	–	0.747
Female	215 (41.7)	46 (21.4)	169 (78.6)	1.07 (0.70-1.64)	
**ICU Stay** (reference: ICU stay is for ≤ 1 week)^**†**^					
ICU stay is for ≤ 1 week	137 (69.2)	69 (50.4)	68 (49.6)	–	0.002^d^
ICU stay is for >1 week	61 (30.8)	45 (73.8)	16 (26.2)	0.36 (0.19-0.70)	
**Concomitant use of antiplatelet** (reference: Single antiplatelet agent)					
Not on antiplatelet therapy	358 (69.3)	74 (20.7)	284 (79.3)	1.26 (0.76-2.09)	0.488
Single antiplatelet therapy	109 (21.1)	27 (24.8)	82 (75.2)	–	
Dual antiplatelet therapy	49 (9.49)	13 (26.5)	36 (73.5)	0.91 (0.42-1.97)	
**Concomitant use of anticoagulant** (reference: Not receiving anticoagulant therapy)†					
Not receiving anticoagulant therapy	361 (70)	85 (23.5)	276 (76.5)	–	0.225
Receiving anticoagulant therapy	155 (30)	29 (18.7)	126 (81.3)	1.34 (0.84-2.14)	

***CI***, confidence interval; ***UOR***, unadjusted odds ratio.

^a^Percentages for the column.

^b^Percentages for the row.

^c^Univariate analysis was conducted to test the associations between variables with treatment appropriateness.

^d^Statistically significant (*P* < 0.05).

^e^Independent t-test was used.

†Retained in the final model.

The clinician in charge was also significantly associated with inappropriate SUP practice (P < 0.001). Patients managed by residents demonstrated significantly higher odds of inappropriate SUP use compared with those managed by general physicians (UOR = 3.11, 95% CI = 1.99–4.86). Length of hospital stay showed a statistically significant association with inappropriate SUP practice (P = 0.003). In addition, ICU stay duration was significantly associated with inappropriate SUP use, where patients with an ICU stay exceeding one week were less likely to experience inappropriate practice compared to patients with ICU stays of one week or less (UOR = 0.36, 95% CI = 0.19–0.70, P = 0.002).

Age group, gender, and concomitant antiplatelet therapy were not significantly associated with inappropriate SUP practice.

Binary logistic regression using a backward stepwise approach identified independent predictors associated with the overall inappropriate use of ASMs for SUP practice (Table 5). Patients receiving anticoagulant therapy were significantly more likely to experience inappropriate SUP practice compared with patients not receiving anticoagulant therapy (AOR = 2.78, 95% CI = 1.35–5.74, P = 0.006).

**Table 5:** Logistic regression analysis of significant predictors associated with overall inappropriate use of acid suppressant medications for stress ulcer prophylaxis practice among study participants (N = 516).

Predictors	UOR	B	SE	Wald	AOR	95% CI	*P*
**Constant**		0.12	0.25	0.266	1.13		0.60
**Concomitant use of anticoagulant** (reference: Not receiving anticoagulant therapy)							
Receiving Anticoagulant Therapy	1.34	1.02	0.36	7.69	2.78	1.35-5.74	0.006^b^

***AOR***, adjusted odds ratio; ***B***, coefficient for the constant in the null model; ***CI***, confidence interval; ***SE***, standard error; ***UOR***, unadjusted odds ratio; ***Wald***, Wald chi-square test that tests the null hypothesis.

^a^Binary logistic regression using backward stepwise analysis.

^b^Statistically significant (*P* < 0.05).

Hosmer–Lemeshow goodness-of-fit test (χ^2^ = 4.574, df = 5, P = 0.470).

## 4. Discussion

This multicenter survey assessed real-world prescribing practices among 552 hospitalized patients across ten Lebanese hospitals. These ten hospitals are from different regions of Lebanon and were included to improve the representativeness and generalizability of the study findings. Overall, this study showed that 90.8% and 77.2% of hospitalized patients did not meet the criteria for SUP according to the SCCM/ASHP 2024 and ASHP 1999 guidelines, respectively. It is concerning that the majority of patients receiving SUP were non-critically ill medical patients, as many of these individuals may not have had a clear indication for prophylaxis based on current evidence-based recommendations. This finding is consistent with the study by Qadeer et al. which evaluated 17,707 patients in a retrospective case-control study and demonstrated that hospital-acquired gastrointestinal bleeding is uncommon among non-critically ill patients, suggesting that routine SUP is unnecessary for most hospitalized patients [18]. Similarly, Heidelbaugh et al. reported that SUP use in non-ICU settings was associated with substantial healthcare expenditures without a corresponding improvement in quality of care [19].

The present study revealed a high prevalence of potentially inappropriate ASM use for SUP, indicating substantial deviation from current evidence-based recommendations. Specifically, more than two-thirds of patients received SUP inappropriately, highlighting a persistent tendency toward SUP overutilization in clinical practice. This finding is consistent with previous national and international studies that have documented widespread inappropriate SUP prescribing. In Lebanon, Zeitoun et al. reported that 67.0% of patients receiving acid-suppressive therapy lacked a SUP indication (95% CI: 64.1–69.9%) [5]. Similarly, inappropriate SUP use was reported in 65.7% of patients in Ethiopia (95% CI: 59.3–71.5%) [1], 72.5% in Jordan (95% CI: 65.8–78.3%) [20], and 73.5% in Sudan (95% CI: 68.4–78.1%) [21], suggesting that inappropriate SUP use remains a common challenge across several healthcare settings. Conversely, lower rate was reported in Saudi Arabia, where 46.0% of patients did not meet the criteria for SUP (95% CI: 41.3–50.7%) [22], indicating comparatively better adherence to prescribing recommendations.

The observed variability in appropriateness rates across studies may be explained by several factors. Differences in healthcare settings, patient populations, and study methodologies can influence prescribing patterns and reported prevalence estimates. In addition, the extent to which international and local guidelines are implemented [6,7,23], the availability of institutional protocols, the presence of medication-review programs, and the effectiveness of prescription auditing systems may significantly affect adherence to SUP recommendations. Furthermore, limited involvement of clinical pharmacists in the medication management process may contribute to inappropriate SUP use, as clinical pharmacists play a critical role in evaluating medication appropriateness, identifying unnecessary therapy, and promoting evidence-based prescribing practices [10,24]. Collectively, these findings highlight the ongoing need for targeted interventions, including educational initiatives, standardized institutional protocols, and regular prescription audits, to optimize SUP prescribing and minimize unnecessary ASM use.

Accordingly, healthcare institutions should consider implementing and enforcing updated ASHP guideline-based protocols for SUP [7] supported by educational initiatives such as workshops, conferences, and continuing professional development programs. Additionally, routine medication review, periodic reassessment of SUP indications, and de-prescribing practices should be encouraged to minimize unnecessary ASM use. Greater integration of clinical pharmacists into multidisciplinary healthcare teams may further improve medication appropriateness through systematic review of therapy, identification of inappropriate prescriptions, and promotion of evidence-based prescribing practices [24].

Regarding the choice of ASMs, PPIs were the only medication class prescribed in our study, with all patients receiving a PPI for SUP. This proportion is substantially higher than that reported in several international studies, including Malaysia (23.9%) [25], Jordan (56.0%) [26], Ireland (79.0%) [27], while being slightly higher than the rate reported in Gondar, Ethiopia (92.7%) [28], and comparable to findings from China (96.1%) [29]. The exclusive use of PPIs in our study likely reflects prescribers’ preference for these agents because of their potent and sustained acid-suppressive effects, and convenient dosing schedules [30].

However, the universal use of PPIs raises concerns regarding potential overreliance on a single therapeutic class, particularly in patients without clear indications for SUP. Although PPIs are generally considered more effective than H2RAs, their widespread and often unnecessary use has been associated with increased healthcare costs and a higher risk of adverse events, including pneumonia, *C. difficile* infection, electrolyte disturbances, and drug-drug interactions [31]. Given that the majority of patients in our study did not meet guideline-based criteria for SUP, the high utilization of PPIs further highlights the need to strengthen adherence to evidence-based prescribing practices.

The most commonly prescribed PPIs in our study were omeprazole (75.4%) and esomeprazole (11.4%). Similar prescribing patterns have been reported in other studies evaluating SUP practices. In a Saudi Arabian study assessing adherence to national SUP guidelines, omeprazole was the predominant PPI prescribed, accounting for approximately 80% of SUP prescriptions, whereas esomeprazole represented only 20% of prescriptions [22]. Comparable findings have also been reported in Lebanon, where omeprazole was the most frequently prescribed PPI (47.5%), followed by rabeprazole (8.6%), lansoprazole (5.0%), and esomeprazole (0.5%) [5].

Regarding the initiated dose of ASMs, the majority of patients received a standard 40 mg dose (92.6%), while only a small proportion were initiated on 20 mg (7.4%). Overall, dose appropriateness was high in our study, with 96.9% of prescriptions considered appropriate and only 3.1% classified as inappropriate. This finding suggests that prescribers in our setting generally adhere well to recommended dosing regimens for PPIs when ASM is initiated, likely reflecting familiarity with standard SUP dosing protocols and the widespread use of fixed-dose regimens such as 40 mg once daily [6,7]. Although, the high rate of appropriate dosing in our study is clinically significant, however this suggests that the main issue in SUP practice is not dose selection but rather overutilization and extended duration of therapy. Standardized dosing practices may reduce variability in prescribing; however, they may also contribute to automatic continuation of therapy without reassessment of clinical need. Consequently, even appropriately dosed PPIs can contribute to medication overuse when prescribed in the absence of guideline-based indications.

These findings highlight an important opportunity for targeted interventions. While dose-related prescribing appears satisfactory, stewardship efforts should prioritize improving appropriate indication assessment and ensuring timely discontinuation of therapy rather than focusing solely on dose adjustments. Implementation of guideline-based order sets [6,7], pharmacist-led medication review, and automatic stop or review dates at ICU transfer may help optimize SUP use [10,24].

In the present study, the intravenous (IV) route was the most commonly used route of administration for SUP, accounting for 87% of patients, followed by the oral route (12%), while enteral administration was rarely used (1%). Current guidelines recommend oral or enteral PPIs whenever feasible, reserving IV administration for patients who cannot tolerate enteral therapy or have impaired gastrointestinal absorption [7]. However, because documentation of patients’ ability to tolerate oral or enteral therapy was not consistently available, the appropriateness of the route could not be reliably determined for all patients. This finding is consistent with studies from Ethiopia, where the IV route was also predominantly used, reaching 97.3% in a study conducted at Debre Tabor Comprehensive Specialized Hospital [11], and approximately 90% in a study conducted at Dessie Referral Hospital [32].

Regarding the duration of ASM therapy, among patients admitted to the ICU, 88.8% received therapy for an appropriate duration, whereas 11.2% continued treatment after the SUP indication had resolved or following ICU transfer without a persisting indication. This finding indicates substantial non-adherence to evidence-based recommendations, which advocate discontinuing SUP once critical illness has resolved and patients are no longer exposed to major risk factors for stress-related mucosal bleeding [6,7]. Our findings are consistent with those reported internationally. In Australia, 63% of patients without risk factors for gastrointestinal bleeding continued SUP following transfer from the ICU [33]. Collectively, these findings demonstrate that the inappropriate continuation of SUP beyond the ICU setting remains a common and persistent challenge across diverse healthcare systems.

The high proportion of patients receiving ASMs beyond their ICU stay observed in this study is clinically significant because prolonged and unnecessary exposure to ASMs may increase the risk of adverse outcomes, including hospital-acquired pneumonia, *C. difficile* infection, drug-drug interactions, micronutrient deficiencies, and increased healthcare costs [31]. These findings highlight an important opportunity for quality improvement initiatives aimed at optimizing SUP prescribing practices. Hospitals should implement clear protocols for the timely discontinuation of ASM once ICU-related indications have resolved. In addition, pharmacist-led medication reviews, electronic stop orders, medication reconciliation at ICU transfer and discharge, and regular prescribing audits may help reduce unnecessary continuation of therapy.

In the present study, only 7.9% were irrationally discharged on ASMs. This rate is significantly lower than the published study conducted in the United States, where 76% of patients continued PPI therapy at hospital discharge [34]. The lower rate observed in our study may reflect differences in prescribing practices, discharge medication reconciliation processes, and growing awareness of the importance of discontinuing unnecessary therapy once the indication has resolved. This finding is consistent with current guideline recommendations [6,7], which advocate discontinuation of SUP when risk factors for gastrointestinal bleeding are no longer present. Nevertheless, inappropriate continuation of ASMs at discharge remains clinically relevant, as medications initiated during hospitalization are often continued long term without reassessment.

The multivariable logistic regression analysis identified anticoagulant therapy as significant predictors of inappropriate ASMs use for SUP. Patients receiving anticoagulant therapy had nearly three times higher odds of inappropriate SUP use compared with those not receiving anticoagulants (P = 0.006). This association may reflect the tendency of clinicians’ perception of anticoagulant therapy as a risk factor for gastrointestinal bleeding, leading to a greater propensity to prescribe SUP.

### 4.1. Study limitations

This is a large multicenter study conducted across ten Lebanese hospitals, providing representation across multiple hospital settings. It provides a comprehensive evaluation of SUP prescribing, including indication, dose, route, duration, and discharge prescribing. Appropriateness was assessed using both ASHP 1999 and SCCM/ASHP 2024 guidelines, allowing comparison with both historical and updated standards. However, this study has several limitations that should be pointed out. First, although prospective in design, its cross-sectional nature limits causal inference between patient characteristics and inappropriate SUP practice. Second, although the route of ASM administration was documented, the study did not specifically assess patients’ ability to tolerate oral or enteral therapy or document the clinical reasons necessitating intravenous administration. Therefore, the appropriateness of the route of administration could not be reliably determined and was not included in the appropriateness assessment. Route of administration was consequently reported descriptively only. Additionally, although participants were recruited from ten hospitals, the statistical analysis did not account for potential clustering of patients within hospitals. Patients treated within the same institution may share similar prescribing practices and institutional characteristics, resulting in correlated observations. This may have led to underestimated standard errors, narrower confidence intervals, and potentially inflated statistical significance of some associations; therefore, the regression findings should be interpreted with caution. Finally, although the study included ten hospitals from different regions of Lebanon, the participating hospitals were not randomly selected and may not fully represent all healthcare institutions in the country. Variations in hospital characteristics, local prescribing policies, and clinical practices may therefore limit the generalizability of the findings to all Lebanese hospitals or to healthcare settings outside Lebanon.

## 5. Conclusions

Inappropriate SUP remains highly prevalent in Lebanese hospitals, particularly regarding indication, duration, and continuation beyond ICU stay or after resolution of the SUP indication. While dosing practices were largely appropriate, excessive PPI use persists, exposing patients to avoidable risks and increased healthcare costs. The presence of anticoagulant therapy independently increased the likelihood of inappropriate use. These findings highlight a persistent gap between guideline recommendations and real-world practice. Targeted stewardship strategies are needed, including standardized protocols, pharmacist-led interventions, clinical decision-support systems, and routine reassessment of SUP necessity. Importantly, the development and implementation of clear, updated guidelines specifically addressing SUP in non-critically ill hospitalized patients are warranted to reduce variability in practice and improve rational prescribing. Future studies should evaluate the impact of such interventions on prescribing quality and patient outcomes.

## Supporting information

S1 Appendix Data collection form. (PDF)

S1 Data De-identified study dataset. De-identified individual-level dataset underlying the findings reported in this study.
(XLSX)
