# Supplementary material for: Assessment of the appropriateness of stress ulcer prophylaxis practice among hospitalized patients across Lebanese hospitals: A multicenter prospective study
Source: PLoS One. 2026 Nov 6;21(11):e0361969. doi: 10.1371/journal.pone.0361969 (PMC13742909; doi:10.1371/journal.pone.0361969)
Supplement: Supplementary file 1 [file pone.0361969.s001.pdf]

Supplementary Appendix. Data Collection Form – SUP Practices

Does the patient have gastrointestinal history that requires PPI intake (including: history of peptic ulcer disease, previous upper gastrointestinal bleeding, erosive esophagitis, Barrett's esophagus, Severe or complicated GERD , H. pylori–associated ulcer disease, Zollinger–Ellison syndrome ,or chronic NSAID or aspirin use in high-risk patients):

- No (continue)
- Yes (Submit survey) This patient is excluded from this form.

If yes, specify:

a. History of peptic ulcer disease
b. Previous upper gastrointestinal bleeding
c. Erosive esophagitis
d. Barrett's esophagus
e. Severe or complicated GERD
f. H. pylori–associated ulcer disease
g. Zollinger–Ellison syndrome
h. Chronic NSAID or antiplatelet use in high-risk patients

Section one: Patient's Sociodemographic Information

1. **Date of case assessment:**
2. **Hospital:**
   a. University-Affiliated
   b. Non-University Affiliated
3. **Patient's Initial :**
4. **Gender :**
   a. Male
   b. Female
5. **Age:**
   Continuous
6. **Admitted unit:**
   a. Internal Medicine (IM)
   b. Intensive Care Unit (ICU)
   c. Cardiac Care Unit (CCU)

7. **Duration of hospitalization since the 1st day of admission:**
8. **Level of SUP prescribers:**
   - **Intern**
   - **Resident**
   - **General physician/hospitalist**
   - **Specialist/attending (branched question)**

9. **Prescriber specialty:**
   - Internal Medicine
   - Gastroenterology
   - Cardiology
   - Pulmonology
   - Nephrology
   - Endocrinology
   - Infectious Diseases
   - Hematology / Oncology
   - Neurology
   - General surgery
   - Others:

Section two: Risk Factors

---

10. **ICU:**
    - No
    - Yes

11. **If the patient is in the ICU, his/her stay is for:**
    - No or ≤ 1 week
    - > 1 week

12. **Coagulopathy (i.e. platelet count of < 50,000 mm^3, INR of ≥ 1.5, or PTT > 2 times the control)**
    - No
    - Yes

13. **On mechanical ventilation for > 48hrs:**
    - No
    - Yes

14. **History of gastrointestinal ulceration or bleeding within 1 year of admission:**
    - No
    - Yes

15. **Glasgow Coma score of ≤ 10, or or inability to obey simple commands:**
   - No
   - Yes

16. **Multiple trauma or traumatic brain injury (injury severity score of ≥ 16):**
   - No
   - Yes

17. **Spinal cord injury:**
   - No
   - Yes

18. **Partial hepatectomy:**
   a. No
   b. Yes

19. **Hepatic failure (chronic liver disease):**
   - No
   - Yes

20. **Thermal injury to > 35% of body surface area:**
   - No
   - Yes

21. **Hepatic or renal transplantation:**
   - No
   - Yes

22. **Patient has shock:**
   - No
   - Yes

23. **Glucocorticoid therapy (>250 mg hydrocortisone or the equivalent):**
   - No
   - Yes

24. **Occult or O=overt GI bleeding lasting ≥ 6 days:**
   - No
   - Yes

25. **NSAIDs intake > 3 month:**
   - No
   - Yes

26. **Antiplatelet intake:**
   - o No
   - o Yes, single antiplatelet agent
   - o Yes, dual antiplatelet therapy

27. **Anticoagulant intake:**
   - o No
   - o Yes

Section three: Assessment of stress ulcer prophylaxis practice

28. Is SUP indicated in this patient as per the guideline (met Criteria for ASHP Guidelines 2024?)
- No
- Yes, critically ill plus coagulopathy, shock, OR chronic liver disease

29. Is SUP indicated in this patient as per the guideline (met Criteria for ASHP Guidelines 1999?)
- No
- Yes (branched)

If yes, why?
- ICU plus One major risk factor
- Non-ICU plus one major risk factor
- ICU plus two minor risk factors
- Non-ICU plus two minor risk factors

30. Based on guideline-defined criteria (ASHP 1999), what was the status of stress ulcer prophylaxis during the patient's hospital stay?
- No indication and no prophylaxis was given (end of the survey)
- No indication but prophylaxis was given
- There is an Indication but no prophylaxis was given (end of the survey)
- There is an Indication and prophylaxis was given

Section four: Evaluation of the appropriateness of PPI/ ASM prescribing practices

31. Acid suppressant Medications (ASM) used:
- Omeprazole
- Esomeprazole
- Pantoprazole
- Lansoprazole
- Dexlansoprazole
- Rabeprazole
- Dexrabeprazole
- Cimetidine
- Famotidine
- Antacids
- Sucralfate
- Misoprostol
- Others: __________

32. Regimen (dose + route + duration) [open ended]

33. Dose initiated:

34. Was the prophylactic **dose** appropriate?
- o No
- o Yes

35. Route of administration:
- o Oral
- o Parenteral (IV)
- o Enteral (gastrostomy/PEG tube/nasogastric)

36. Was the route of administration appropriate?
- o No
- o Yes

37. Duration of ASM (in days):

38. Was the duration of AST appropriate?
- o No, exceeding the ICU stay
- o Yes, only during the ICU stay

39. Was the patient discharged on ASM?
- o No
- o Yes

40. Overall, the SUP practice for this patient is: (indication, dose, route, and duration)
- o Appropriate
- o Inappropriate

Section five: Consequences

---

41. Did the patient experience any of the following adverse effects after starting ASM therapy?
- o No
- o Yes, *Clostridioides difficile* infection
- o Yes, Pneumonia
- o Yes, Drug interactions
- o Yes, others
