# Supplementary material for: Assessment of the appropriateness of stress ulcer prophylaxis practice among hospitalized patients across Lebanese hospitals: A multicenter prospective study
Source: PLoS One. 2026 Nov 6;21(11):e0361969. doi: 10.1371/journal.pone.0361969 (PMC13742909; doi:10.1371/journal.pone.0361969)
Supplement: Peer review history

# Peer review: Decision Letter 0

**DOI:** 10.1371/journal.pone.0361969.r001

**Authors:** Jad El Masri

16 Aug 2026

Dear Dr. Itani,

Thank you for submitting your manuscript to PLOS ONE. After careful consideration, we feel that it has merit but does not fully meet PLOS ONE’s publication criteria as it currently stands. Therefore, we invite you to submit a revised version of the manuscript that addresses the points raised during the review process.

Please submit your revised manuscript by Oct 15 2026 11:59PM. If you will need more time than this to complete your revisions, please reply to this message or contact the journal office at plosone@plos.org. When you're ready to submit your revision, log on to https://www.editorialmanager.com/pone/ and select the 'Submissions Needing Revision' folder to locate your manuscript file.

- A letter that responds to each point raised by the academic editor and reviewer(s). You should upload this letter as a separate file labeled 'Response to Reviewers'.
- A marked-up copy of your manuscript that highlights changes made to the original version. You should upload this as a separate file labeled 'Revised Manuscript with Track Changes'.
- An unmarked version of your revised paper without tracked changes. You should upload this as a separate file labeled 'Manuscript'.

If you would like to make changes to your financial disclosure, please include your updated statement in your cover letter. Guidelines for resubmitting your figure files are available below the reviewer comments at the end of this letter.

If applicable, we recommend that you deposit your laboratory protocols in protocols.io to enhance the reproducibility of your results. Protocols.io assigns your protocol its own identifier (DOI) so that it can be cited independently in the future. For instructions see: https://journals.plos.org/plosone/s/submission-guidelines#loc-laboratory-protocols. Additionally, PLOS ONE offers an option for publishing peer-reviewed Lab Protocol articles, which describe protocols hosted on protocols.io. Read more information on sharing protocols at https://plos.org/protocols?utm_medium=editorial-email&utm_source=authorletters&utm_campaign=protocols.

As the corresponding author, your ORCID iD is verified in the submission system and will appear in the published article. PLOS supports the use of ORCID, and we encourage all coauthors to register for an ORCID iD and use it as well. Please encourage your coauthors to verify their ORCID iD within the submission system before final acceptance, as unverified ORCID iDs will not appear in the published article. *Only*  the individual author can complete the verification step; PLOS staff *cannot*  verify ORCID iDs on behalf of authors.

We look forward to receiving your revised manuscript.

Kind regards,

Jad El Masri

Academic Editor

PLOS One

Journal Requirements:

When submitting your revision, we need you to address these additional requirements.

1. Please ensure that your manuscript meets PLOS ONE's style requirements, including those for file naming. The PLOS ONE style templates can be found at

https://journals.plos.org/plosone/s/file?id=wjVg/PLOSOne_formatting_sample_main_body.pdf and https://journals.plos.org/plosone/s/file?id=ba62/PLOSOne_formatting_sample_title_authors_affiliations.pdf

2. Your ethics statement should only appear in the Methods section of your manuscript. If your ethics statement is written in any section besides the Methods, please delete it from any other section.

3. In the online submission form, you indicated that the datasets generated and analyzed during the current study are not publicly available due to ethical and confidentiality restrictions, but are available from the corresponding author upon reasonable request and with permission from the BAU IRB and ethical committees of the participating institutions. The data contain potentially identifying or sensitive information, which could risk patient and hospital re-identification. Additionally, given that this study assessed hospital management practices, further confidentiality concerns apply. In accordance with ethical standards, access to these datasets requires a formal request to the corresponding author (r.itani@bau.edu.lb) or to the IRB at Beirut Arab University (irb@bau.edu.lb). All relevant data supporting the conclusions of this study are included within the article. Furthermore, the data will be securely stored in Beirut Arab University’s institutional repository, ensuring long-term availability and accessibility through the IRB.

All PLOS journals now require all data underlying the findings described in their manuscript to be freely available to other researchers, either 1. In a public repository, 2. Within the manuscript itself, or 3. Uploaded as supplementary information.

This policy applies to all data except where public deposition would breach compliance with the protocol approved by your research ethics board. If your data cannot be made publicly available for ethical or legal reasons (e.g., public availability would compromise patient privacy), please explain your reasons on resubmission and your exemption request will be escalated for approval.

4. Please include captions for your Supporting Information files at the end of your manuscript, and update any in-text citations to match accordingly. Please see our Supporting Information guidelines for more information: http://journals.plos.org/plosone/s/supporting-information.

6. If the reviewer comments include a recommendation to cite specific previously published works, please review and evaluate these publications to determine whether they are relevant and should be cited. There is no requirement to cite these works unless the editor has indicated otherwise.

[Note: HTML markup is below. Please do not edit.]

Reviewers' comments:

Reviewer's Responses to Questions

**Comments to the Author**

1. Is the manuscript technically sound, and do the data support the conclusions?

Reviewer #1: Yes

Reviewer #2: Partly

**********

2. Has the statistical analysis been performed appropriately and rigorously?

Reviewer #1: Yes

Reviewer #2: No

**********

3. Have the authors made all data underlying the findings in their manuscript fully available?

The PLOS Data policy

Reviewer #1: Yes

Reviewer #2: No

**********

4. Is the manuscript presented in an intelligible fashion and written in standard English?

Reviewer #1: Yes

Reviewer #2: Yes

**********

Reviewer #1: This manuscript addresses an important clinical issue regarding the appropriateness of stress ulcer prophylaxis prescribing in Lebanese hospitals. The study provides valuable data and is generally well structured. However, several methodological, statistical, and reporting issues require clarification before the manuscript can be considered for publication.

Comments:

1. please provide more detailed information about the participating hospitals. This would improve transparency and allow readers to better assess the representativeness of the sample.

2. There is an inconsistency in the description of the multivariable logistic regression procedure. The methods state that a backward stepwise selection procedure was used, whereas the Table 5 footnote states that forward stepwise analysis was performed.

3.remover subsection operational definition.

4. minimize subsection ethical considerations.

5. minimize the discussion.

Overall, the study addresses a relevant clinical issue and provides useful findings. However, several methodological and reporting concerns, including study design, statistical analysis, data consistency, and generalizability, should be addressed. I therefore recommend **minor revision**.

Reviewer #2: This is a relevant multicenter study addressing an important medication-stewardship issue. The inclusion of ten hospitals and the comparison of prescribing practices against both the 1999 and 2024 guidelines are valuable strengths. However, in my opninion, several methodological and reporting inconsistencies should be resolved before the findings can be interpreted confidently.

+Major comments

- The study population and denominators require clarification. The Methods indicate that eligible patients were adults prescribed acid-suppressive medication for stress ulcer prophylaxis, yet 552 patients were enrolled and only 516 received prophylaxis. Please explain how the remaining 36 patients entered the cohort and provide a clear patient flow showing the numbers screened, excluded, treated, and analyzed.

- The indication counts are inconsistent. The manuscript reports 126 patients eligible under the 1999 guideline and 51 under the 2024 guideline, whereas Figure 1 classifies 136 patients as having an indication. Please reconcile these totals and state clearly which guideline, or combination of guidelines, was used for Figure 1 and for the overall appropriateness outcome.

- Duration appropriateness appears to have been assessed according to whether treatment continued beyond the ICU stay for all 516 treated patients. However, many participants were admitted only to Internal Medicine or CCU wards. I suggest analyzing ICU and non-ICU patients separately and using a clinically relevant duration criterion for patients who were never admitted to the ICU.

- Mechanical ventilation is used as a predictor of inappropriate prophylaxis, but it is also part of the criteria used to determine appropriateness under the 1999 guideline. This creates a circular relationship between the predictor and outcome and likely explains the very low adjusted odds ratio. Mechanical ventilation should not be presented as an independent predictor without directly addressing this issue. A sensitivity analysis using an outcome definition that does not incorporate the predictor would be helpful.

- The statistical methods describe backward stepwise selection, while the footnote to Table 5 states that forward stepwise analysis was used. Please clarify which method was actually applied and revise the manuscript accordingly. The complete regression model, including all variables entered, model-fit results, and missing-data handling, should also be reported.

- Because participants were recruited from ten hospitals, observations within the same hospital may be correlated. Ideally, the analysis should account for hospital-level clustering, for example through a mixed-effects model or cluster-robust standard errors. If this cannot be done, the possible effect on the confidence intervals and statistical significance should at least be acknowledged as a limitation.

- The claim that all routes of administration were appropriate requires further support, particularly because 87% of patients received intravenous therapy. Please describe the criteria used to determine route appropriateness and how the ability to tolerate oral or enteral treatment was assessed. Where this information was unavailable, route appropriateness may need to be classified as uncertain rather than appropriate.

- The data-availability statement should be strengthened. The manuscript states that no patient identifiers were recorded, but the dataset is available only upon request because of confidentiality and re-identification concerns. Please explain more specifically why a de-identified minimal dataset cannot be shared publicly. If access must remain restricted, objective access conditions and an institutional contact responsible for reviewing requests should be provided.

- Pneumonia and drug interactions are described as adverse effects associated with acid-suppressive therapy. Because this observational study does not appear to include a comparator or formal causality assessment, these events should not be attributed directly to the medications. Please provide the definitions and assessment methods used or describe them more cautiously as events observed during treatment.

+ Minor comments. Kindly:

- Correct the garbled inappropriate-practice entry in Table 3, which appears to be 402 (77.9%).

- Recalculate the route percentages in Table 3; for example, 56 of 516 is approximately 10.9%, not 12%.

- Correct the Nephrology entry in Table 4, which presumably should be 1 (0.2%).

- Replace the Table 4 footnote referring to “intervention acceptance,” as this is unrelated to the study outcome.

- Standardize the use of ASM and AST throughout the manuscript.

- Correct the duplicate subsection number “2.7.”

- Reconcile the statement that all eligible patients had an ICU stay with the reported ward distribution and guideline-specific eligibility counts.

- Correct typographical and grammatical errors, including “Parentral,” “Data collectors Training,” and the unmatched quotation mark after the SPSS version.

- Consider reporting confidence intervals for the principal prevalence estimates.

- Moderate statements about increased costs and adverse effects, as these outcomes were not directly evaluated in this study.

Overall, this study has the potential to provide useful information on stress ulcer prophylaxis practices in Lebanon. Addressing the inconsistencies in the cohort definition, guideline classification, duration assessment, and statistical analysis would substantially improve the reliability and clarity of the manuscript.

**********

what does this mean?). If published, this will include your full peer review and any attached files.

If you choose “no”, your identity will remain anonymous but your review may still be made public.

**Do you want your identity to be public for this peer review?**  For information about this choice, including consent withdrawal, please see our Privacy Policy

Reviewer #1: No

Reviewer #2: No

**********

[NOTE: If reviewer comments were submitted as an attachment file, they will be attached to this email and accessible via the submission site. Please log into your account, locate the manuscript record, and check for the action link "View Attachments". If this link does not appear, there are no attachment files.]

To ensure your figures meet our technical requirements, please review our figure guidelines: https://journals.plos.org/plosone/s/figures

You may also use PLOS’s free figure tool, NAAS, to help you prepare publication quality figures: https://journals.plos.org/plosone/s/figures#loc-tools-for-figure-preparation.

NAAS will assess whether your figures meet our technical requirements by comparing each figure against our figure specifications.

# Author comment: Author response to Decision Letter 1

**DOI:** 10.1371/journal.pone.0361969.r002

8 Oct 2026

Dear Prof. Jad El Masri,

Academic Editor

PLOS ONE

Submission ID: PONE-D-26-27216

Thank you for giving us the opportunity to submit a revised draft of our manuscript titled “Assessment of the Appropriateness of Stress Ulcer Prophylaxis Practice among Hospitalized Patients across Lebanese Hospitals: A Multicenter Prospective Study” to PLOS One. We appreciate the time and effort that you and the reviewers have dedicated to providing valuable feedback on our manuscript. We are grateful to your insightful comments. We have been able to incorporate changes to reflect all of the suggestions you and the reviewers have provided. Those changes are highlighted within the manuscript.

Please see below, in blue, for point-by-point responses to the comments and concerns

Editor’s comments:

Journal Requirements:

When submitting your revision, we need you to address these additional requirements.

1- Please ensure that your manuscript meets PLOS ONE's style requirements, including those for file naming. The PLOS ONE style templates can be found at

https://journals.plos.org/plosone/s/file?id=wjVg/PLOSOne_formatting_sample_main_body.pdf and https://journals.plos.org/plosone/s/file?id=ba62/PLOSOne_formatting_sample_title_authors_affiliations.pdf

Authors’ response: Thank you for this important reminder. We carefully reviewed the PLOS ONE style requirements and reformatted the manuscript accordingly. Specifically, we revised the title page, author affiliations, section headings, page numbering, continuous line numbering, spacing, table formatting, figure citations and captions, and reference citation style to align with the journal’s guidelines. All main headings and subheadings were revised to sentence case, and tables were retained as editable tables and placed directly after their first citation in the text.

2- Your ethics statement should only appear in the Methods section of your manuscript. If your ethics statement is written in any section besides the Methods, please delete it from any other section.

Author’s response:

Thank you for this important comment. We confirm that the ethics statement has been retained within the Methods section under the subsection “Ethical considerations.” Any duplicate ethics statement appearing elsewhere in the manuscript, including the end-matter declarations, has been removed.

3- In the online submission form, you indicated that the datasets generated and analyzed during the current study are not publicly available due to ethical and confidentiality restrictions, but are available from the corresponding author upon reasonable request and with permission from the BAU IRB and ethical committees of the participating institutions. The data contain potentially identifying or sensitive information, which could risk patient and hospital re-identification. Additionally, given that this study assessed hospital management practices, further confidentiality concerns apply. In accordance with ethical standards, access to these datasets requires a formal request to the corresponding author (r.itani@bau.edu.lb) or to the IRB at Beirut Arab University (irb@bau.edu.lb). All relevant data supporting the conclusions of this study are included within the article. Furthermore, the data will be securely stored in Beirut Arab University’s institutional repository, ensuring long-term availability and accessibility through the IRB.

All PLOS journals now require all data underlying the findings described in their manuscript to be freely available to other researchers, either 1. In a public repository, 2. Within the manuscript itself, or 3. Uploaded as supplementary information. This policy applies to all data except where public deposition would breach compliance with the protocol approved by your research ethics board. If your data cannot be made publicly available for ethical or legal reasons (e.g., public availability would compromise patient privacy), please explain your reasons on resubmission and your exemption request will be escalated for approval.

Author’s response: In response to this comment, we consulted IRB at BAU regarding the sharing of the study data. We received oral approval from the IRB to share the de-identified dataset, provided that no medical record numbers, patient identifiers, or hospital identifiers are disclosed.

Accordingly, we have revised the data availability approach. The dataset underlying the findings of this study has now been fully de-identified and provided as an Excel file in the Supplementary Appendix. The supplementary dataset does not contain medical record numbers, patient-identifying information, or hospital identifiers, thereby minimizing any risk of patient or hospital re-identification.

The supplementary dataset has also been appropriately cited within the manuscript to ensure transparency and facilitate access to the data supporting the study findings. The Data Availability Statement now reads: “All relevant data supporting the findings of this study are available within the manuscript and its Supporting Information files.”

4- Please include captions for your Supporting Information files at the end of your manuscript, and update any in-text citations to match accordingly. Please see our Supporting Information guidelines for more information: http://journals.plos.org/plosone/s/supporting-information.

Authors’ response: Thank you for this comment. We have added captions for all Supporting Information files at the end of the revised manuscript and updated the corresponding in-text citations accordingly.

5- If the reviewer comments include a recommendation to cite specific previously published works, please review and evaluate these publications to determine whether they are relevant and should be cited. There is no requirement to cite these works unless the editor has indicated otherwise.

Authors’ response: We have not been asked by the reviewers’ to cite any research paper.

[Note: HTML markup is below. Please do not edit.]

Authors’ response: The reviewers did not recommend or request the citation of any additional references.

Reviewers' comments:

Reviewer's Responses to Questions

Comments to the Author

1. Is the manuscript technically sound, and do the data support the conclusions?

The manuscript must describe a technically sound piece of scientific research with data that supports the conclusions. Experiments must have been conducted rigorously, with appropriate controls, replication, and sample sizes. The conclusions must be drawn appropriately based on the data presented.

Reviewer #1: Yes

Reviewer #2: Partly

________________________________________

2. Has the statistical analysis been performed appropriately and rigorously?

Reviewer #1: Yes

Reviewer #2: No

________________________________________

3. Have the authors made all data underlying the findings in their manuscript fully available?

The PLOS Data policy requires authors to make all data underlying the findings described in their manuscript fully available without restriction, with rare exception (please refer to the Data Availability Statement in the manuscript PDF file). The data should be provided as part of the manuscript or its supporting information, or deposited to a public repository. For example, in addition to summary statistics, the data points behind means, medians and variance measures should be available. If there are restrictions on publicly sharing data—e.g. participant privacy or use of data from a third party—those must be specified.

Reviewer #1: Yes

Reviewer #2: No

Authors’ response: We have now made the complete de-identified dataset underlying the findings of this study available as a Supporting Information file (S1 Dataset). The dataset has also been appropriately cited in the revised manuscript.

________________________________________

4. Is the manuscript presented in an intelligible fashion and written in standard English?

PLOS ONE does not copyedit accepted manuscripts, so the language in submitted articles must be clear, correct, and unambiguous. Any typographical or grammatical errors should be corrected at revision, so please note any specific errors here.

Reviewer #1: Yes

Reviewer #2: Yes

________________________________________

Reviewers’ comments:

Please use the space provided to explain your answers to the questions above. You may also include additional comments for the author, including concerns about dual publication, research ethics, or publication ethics. (Please upload your review as an attachment if it exceeds 20,000 characters)

Reviewer #1:

1- This manuscript addresses an important clinical issue regarding the appropriateness of stress ulcer prophylaxis prescribing in Lebanese hospitals. The study provides valuable data and is generally well structured. However, several methodological, statistical, and reporting issues require clarification before the manuscript can be considered for publication.

Authors’ response: Thank you for your time and effort in reviewing our manuscript and for your valuable feedback. We have carefully addressed all your comments, which have helped improve and enrich the quality of our manuscript.

Comments:

1. please provide more detailed information about the participating hospitals. This would improve transparency and allow readers to better assess the representativeness of the sample.

Author’s response:

Thank you for this important comment. We agree that providing additional information about the participating hospitals would improve the transparency of the study and allow readers to better understand the characteristics and representativeness of the study setting. Accordingly, we have expanded the description of the participating hospitals in the Methods section. The revised text now provides additional information regarding the number and type of participating hospitals, their healthcare sector, level of care, patient populations, and geographic distribution, while maintaining institutional confidentiality and not identifying individual hospitals where this was not permitted. The modified section is as follows:

“The study was conducted across ten large tertiary-care teaching hospitals in Lebanon, including nine private hospitals and one public hospital. The participating hospitals were geographically distributed across three major urban centers: seven hospitals were located in Beirut, the capital of Lebanon; two were located in Saida, the capital of South Lebanon; and one was located in Tripoli, the capital of North Lebanon. The participating hospitals were large healthcare institutions providing tertiary-level and specialized medical services to diverse patient populations. The private hospitals served patients from varied socioeconomic backgrounds, whereas the public hospital primarily served individuals from low- and middle-income groups. Collectively, these institutions provided care across multiple medical specialties and served diverse patient populations, providing a broad study setting for assessing the SUP practice. To maintain institutional confidentiality, individual hospitals are not identified.”

2. There is an inconsistency in the description of the multivariable logistic regression procedure. The methods state that a backward stepwise selection procedure was used, whereas the Table 5 footnote states that forward stepwise analysis was performed.

Author’s response: Thank you for pointing out this inconsistency. The backward stepwise selection procedure was the procedure actually used for the multivariable logistic regression analysis. The reference to “forward stepwise analysis” in the Table 5 footnote was an error.

We have corrected the Table 5 footnote to state “backward stepwise analysis” and reviewed the manuscript to ensure consistency in the description of the statistical analysis throughout. The description of the multivariable logistic regression procedure was further detailed in the methods section as follows:

“Variables with a P-value < 0.25 in univariate testing were considered for inclusion in the multivariable logistic regression model. The variables entered into the initial multivariable model were hospital affiliation status (including university-affiliated vs. non-university-affiliated hospital), clinician in charge, length of hospital stay, ICU stay (including ICU ≤ 1 week vs > 1 week), and receipt of anticoagulant therapy (yes vs. no).

Multicollinearity was evaluated using variance inflation factors (VIFs), with a cut-off value of 10 for exclusion. All VIFs were below 2.5, indicating no evidence of significant multicollinearity among predictors. Variables were further assessed for clinical relevance, with conceptually related factors reviewed for potential inclusion.

The final multivariable logistic regression model was constructed using a backward stepwise selection procedure, in which all candidate variables were initially entered into the model and subsequently removed in a stepwise manner according to their statistical contribution. Model fit was assessed using the Hosmer–Lemeshow goodness-of-fit test. The final model demonstrated adequate fit (χ² = 4.574, df = 5, P = 0.470). Adjusted odds ratios (AORs), along with 95% confidence intervals (CIs) and P-values, were reported for variables retained in the final model. Statistical significance was set at P < 0.05.”

3. Remove the subsection operational definition.

Author’s response: Thank you for the comment. The “Operational Definition” subsection has been removed from the manuscript as requested. The definition and criteria used to assess the appropriateness of SUP practices have been incorporated into the Methods section under the “Data Collection Form” subsection (Section 3), where the assessment approach is described in detail.

4. Minimize subsection ethical considerations.

Author’s response: Thank you for the comment. The Ethical Considerations subsection has been revised and substantially shortened while retaining the essential information regarding ethical approval, confidentiality, data protection, and the waiver of informed consent, as follows:

“2.6. Ethical considerations

This study adhered to the ethical standards outlined in the World Medical Association’s Declaration of Helsinki (19). Ethical approval was obtained from the Institutional Review Board (IRB) at Beirut Arab University on April 16, 2026; no approval code was issued. The IRB confirmed that the study was approved in accordance with institutional regulations and that data collection could proceed. As the study involved prospective review of medical records without direct patient contact, no patient identifiers were collected, and all data were handled confidentially. Access was restricted to the principal investigators, with secure storage of electronic and physical records. Only anonymized datasets were shared with participating institutions. A waiver of informed consent was granted by the IRB due to the observational nature of the study and absence of direct patient interaction.”

5. minimize the discussion.

Author’s response: Thank you for this comment. We have revised and condensed the Discussion section by removing redundant information and shortening the comparison with previous studies, while retaining the key findings and their clinical implications.

Overall, the study addresses a relevant clinical issue and provides useful findings. However, several methodological and reporting concerns, including study design, statistical analysis, data consistency, and generalizability, should be addressed. I therefore recommend **minor revision**.

Authors’ response: Thank you for the positive assessment and constructive comments. We have carefully addressed all methodological, statistical, and reporting concerns raised and revised the manuscript accordingly to improve its clarity, transparency, and overall quality. We have also further acknowledged and discussed the potential limitations related to the generalizability of our findings in the Study Limitations section. We sincerely appreciate your valuable comments, which have helped strengthen the manuscript.

Modified study limitations:

“Study Limitations

This is a large multicenter study conducted across ten Lebanese hospitals, providing representation across multiple hospital settings. It provides a comprehensive evaluation of SUP prescribing, including indication, dose, route, duration, and discharge prescribing. Appropriateness was assessed using both ASHP 1999 and SCCM/ASHP 2024 guidelines, allowing comparison with both historical and updated standards. However, this study has several limitations that should be pointed out. First, although prospective in design, its cross-sectional nature limits causal inference between patient characteristics and inappropriate SUP practice. Second, although the route of ASM administration was documented, the study did not specifically assess patients’ ability to tolerate oral or enteral therapy or document the clinical reasons necessitating intravenous administration. Therefore, the appropriateness of the route of administration could not be reliably determined and was not included in the appropriateness assessment. Route of administration was consequently reported descriptively only. Additionally, although participants were recruited from ten hospitals, the statistical analysis did not account for potential clustering of patients within hospitals. Patients treated within the same institution may share similar prescribing practices and institutional characteristics, resulting in correlated observations. This may have led to underestimated standard errors, narrower confidence intervals, and potentially inflated statistical significance of some associations; therefore, the regression findings should be interpreted with caution. Finally, although the study included ten hospitals from different regions of Lebanon, the participating hospitals were not randomly selected and may not fully represent all healthcare institutions in the country. Variations in hospital characteristics, local prescribing policies, and clinical practices may therefore limit the generalizability of the findings to all Lebanese hospitals or to healthcare settings outside Lebanon.”

Reviewer #2:

This is a relevant multicenter study addressing an important medication-stewardship issue. The inclusion of ten hospitals and the comparison of prescribing practices against both the 1999 and 2024 guidelines are valuable strengths. However, in my opinion, several methodological and reporting inconsistencies should be resolved before the findings can be interpreted confidently.

Authors’ response: Thank you for the positive assessment of our study and for recognizing its clinical relevance and key strengths. We appreciate the valuable and constructive comments and have carefully addressed the methodological and reporting inconsistencies raised. The manuscript has been revised accordingly to improve the clarity, consistency, and interpretability of the findings. We hope that the revised manuscript meets your expectations for publication.

+Major comments

1- The study population and denominators require clarification. The Methods indicate that eligible patients were adults prescribed acid-suppressive medication for stress ulcer prophylaxis, yet 552 patients were enrolled and only 516 received prophylaxis. Please explain how the remaining 36 patients entered the cohort and provide a clear patient flow showing the numbers screened, excluded, treated, and analyzed.

Author’s response: Thank you for highlighting this important point. We have revised the Methods section to clarify the study population and eligibility criteria. All adult patients admitted to the participating units during the study period were assessed for the appropriateness of SUP practice, regardless of whether an acid-suppressive medication was prescribed. This approach was necessary to capture the full spectrum of SUP practice, including appropriate use, inappropriate overutilization, appropriate non-use, and potential underutilization (i.e., patients with an indication for SUP who did not receive prophylaxis).

Accordingly, the overall study cohort comprised 552 patients, of whom 516 received an ASM during hospitalization, irrespective of whether its use was clinically indicated, while 36 did not receive an ASM. The subgroup of 516 ASM recipients was subsequently evaluated for ASM prescribing patterns and appropriateness, including medication selection, dose, and duration of use.

To further clarify the study population, denominators, and assessment process for readers, we have added two flowcharts: one illustrating the overall included study population and the assessment of SUP indication, and another detailing the evaluation of prescribing patterns and appropriateness among patients who received ASMs. The corresponding Methods section has also been revised to clearly describe these populations and assessments, as follows:

“All adults admitted to these units during the study period were assessed for the appropriateness of SUP practice, regardless of whether ASMs were prescribed. This enabled the assessment of both appropriate and potentially inappropriate use, as well as potential underuse of SUP.”

2- The indication counts are inconsistent. The manuscript reports 126 patients eligible under the 1999 guideline and 51 under the 2024 guideline, whereas Figure 1 classifies 136 patients as having an indication. Please reconcile these totals and state clearly which guideline, or combination of guidelines, was used for Figure 1 and for the overall appropriateness outcome.

Author’s response: Thank you for highlighting this point. We have revised the Methods and Figure 1 to clarify how the guideline-based indications were combined. The reported numbers of patients meeting the 1999 ASHP criteria (n = 126) and the 2024 SCCM/ASHP criteria (n = 51) are not mutually exclusive, as some patients met the eligibility criteria of both guidelines. Therefore, these counts should not be summed. After accounting for this overlap, 136 unique patients met the criteria for SUP according to at least one of the two guidelines and were consequently classified as having a clinical indication for SUP in Figure 1.

To ensure consistency, we have added an operational definition of the appropriateness of SUP use based on clinical indication to the Methods. For the combined assessment, a patient was classified as having an indication for SUP if the eligibility criteria of either the 1999 ASHP or the 2024 SCCM/ASHP guideline were met. Accordingly, SUP use was considered appropriate when prophylaxis was prescribed in the presence of an indication according to either guideline, or when prophylaxis was not prescribed in the absence of an indication according to both guidelines. Conversely, SUP use was considered inappropriate when prophylaxis was prescribed despite the absence of an indication according to both guidelines (overutilization) or was not prescribed despite the presence of an indication according to either guideline (underutilization). The following was added to the methods section: “Appropriateness of SUP use based on clinical indication was defined as the prescription of SUP to a patient with an eligible clinical indication according to either the 1999 ASHP or 2024 SCCM/ASHP guidelines, or the absence of SUP prescription when no indication was identified according to either guideline. Conversely, SUP use was considered inappropriate when prophylaxis was prescribed despite the absence of an indication according to both guidelines (overutilization) or when prophylaxis was not prescribed despite the presence of an indication according to either guideline (underutilization) (6,7).”

Figure 1 has also been revised to explicitly illustrate this classification approach and facilitate interpretation of the indication-based appropriateness assessment.

3- Duration appropriateness appears to have been assessed according to whether treatment continued beyond the ICU stay for all 516 treated patients. However, many participants were admitted only to Internal Medicine or CCU wards. I suggest analyzing ICU and non-ICU patients separately and using a clinically relevant duration criterion for patients who were never admitted to the ICU.

Author’s response:

Thank you for this important comment. We agree that applying ICU stay as the criterion for assessing duration appropriateness to all patients receiving an ASM was not appropriate, particularly because a substantial proportion of the study population was managed exclusively in IMU or CCU wards. We have therefore revised the analysis and assessed ASM duration separately according to ICU admission status.

Among the 516 patients who received ASM, 197 had an ICU admission. In this subgroup, duration appropriateness was assessed according to whether ASM was discontinued when the SUP indication resolved or at ICU transfer when prophylaxis was no longer indicated. Of these patients, 175 had an appropriate duration, whereas 22 continued ASM after resolution of the indication or following ICU transfer without a persisting indication and were therefore classified as having an inappropriate duration.

The remaining 319 patients were managed exclusively in Internal Medicine or CCU wards and had no guideline-defined indication for SUP. Accordingly, ASM use in these patients was considered inappropriate from initiation rather than being classified according to ICU-based duration criteria. No non-ICU patient receiving ASM had a guideline-defined indication for SUP.

Table 3 and the corresponding Methods, Results, and Discussion sections have been revised.

The results section was modified as follows: “The mean duration of ASM use was 6.05 ± 11.04 days, ranging from 1 to 61 days. Among the 197 patients admitted to the ICU, ASM duration was considered appropriate in 175 (88.8%), as therapy was discontinued when the SUP indication resolved or at ICU transfer when no longer indicated, whereas 22 (11.2%) had an inappropriate duration due to continuation after resolution of the indication or following ICU transfer without a persisting indication. Among the 319 patients who were not admitted to the ICU, all received ASM without a guideline-defined indication for SUP and were therefore considered to have inappropriate therapy from initiation rather than inappropriate duration.”

4- Mechanical ventilation is used as a predictor of inappropriate prophylaxis, but it is also part of the criteria used to determine appropriateness under the 1999 guideline. This creates a circular relationship between the predictor and outcome and likely explains the very low adjusted odds ratio. Mechanical ventilation should not be presented as an independent predictor without directly addressing this issue. A sensitivity analysis using an outcome definition that does not incorporate the predictor would be helpful.

Author’s response:

Thank you for this important observation. We agree with you that including mechanical ventilation as a predictor of inappropriate SUP practice may introduce a circular relationship, as mechanical ventilation is itself one of the criteria used to determine SUP appropriateness according to the 1999 ASHP guideline. Therefore, we have removed mechanical ventilation from both the univariate and multivariable regression analyses and redesigned the multivariable logistic regression model accordingly. The corresponding Abstract, Methods, Results, tables, and Discussion have also been revised to reflect the updated analysis. We thank you for highlighting this important methodological issue, which helped improve the robustness of our analysis.

5- The statistical methods describe backward stepwise selection, while the footnote to Table 5 states that forward stepwise analysis was used. Please clarify which method was actually applied and revise the manuscript accordingly. The complete regression model, including all variables entered, model-fit results, and missing-data handling, should also be reported.

Author’s response:

Thank you for highlighting this inconsistency. We apologize for the discrepancy between the Statistical Analysis section and the footnote of Table 5. The multivariable logistic regression was performed using a backward stepwise selection method, and the reference to forward stepwise analysis in Table 5 was an error. This has been corrected throughout the manuscript.

In accordance with the reviewer’s recommendation, we have also expanded the description of the multivariable regression analysis to report all variables initially entered into the model, the model-fit assessment, and the handling of missing data. The final regression model demonstrated adequate goodness of fit according to the Hosmer–Lemeshow test (χ² = 4.574, df = 5, P = 0.470). The Statistical Analysis section, Results, and Table 5 and its footnote have been revised accordingly.

Details related to the designing of the regression model were documented in the methods section as follows:

“2.7. Data preparation and analysis

Upon completion of data collection, all submitted forms underwent quality review to assess completeness, readability, internal consistency, and clarity. Each recorded case was verified by the corresponding faculty preceptor. Forms containing missing information, unclear entries, or inconsistent responses were excluded from the analysis. Consequently, no missing data were present in the final dataset used for the statistical analyses.

Data analysis was performed using the IBM Statistical Package for Social Sciences (SPSS®) version ‎‎26.0 (IBM Corp., Armonk, NY, USA). Descriptive statistics were reported as frequencies and percentages for categorical variables and as means with standard deviations for continuous variables. Univariate analyses were performed to examine the association between potential predictors and inappropriate SUP practice using Pearson’s chi-square (χ²) test for categorical variables, and independent t-test for continuous variables. Variables with a P-value < 0.25 in univariate testing were considered for inclusion in the multivariable logistic regression model. The variables entered into the initial multivariable model were hospital affiliation status (including university-affiliated vs. non-university-affiliated hospital), clinician in charge, length of hospital stay, ICU stay (including ICU ≤ 1 week vs > 1 week), and receipt of anticoagulant therapy (yes vs. no).

Multicollinearity was evaluated using variance inflation factors (VIFs), with a cut-off value of 10 for exclusion. All VIFs were below 2.5, indicating no evidence of significant multicollinearity among predictors. Variables were further assessed for clinical relevance, with conceptually related factors reviewed for potential inclusion.

The final multivariable logistic regression model was constructed using a backward stepwise selection procedure, in which all candidate variables were initially entered into the model and subsequently removed in a stepwise manner according to their statistical contribution. Model fit was assessed using the Hosmer–Lemeshow goodness-of-fit test. The final model demonstrated adequate fit (χ² = 4.574, df = 5, P = 0.470). Adjusted odds ratios (AORs), along with 95% confidence intervals (CIs) and P-values, were reported for variables retained in the final model. Statistical significance was set at P < 0.05.”

6- Because participants were recruited from ten hospitals, observations within the same hospital may be correlated. Ideally, the analysis should account for hospital-level clustering, for example through a mixed-effects model or cluster-robust standard errors. If this cannot be done, the possible effect on the confidence intervals and statistical significance should at least be acknowledged as a limitation.

Author’s response: Thank you for this important comment. We agree that, given the multicenter nature of the study, observations from patients within the same hospital may be correlated due to shared institutional characteristics and prescribing practices. The present analysis did not account for hospital-level clustering using mixed-effects models or cluster-robust standard errors. We have therefore acknowledged this issue as a limitation of the study. Specifically, failure to account for within-hospital correlation may have resulted in underestimated standard errors and consequently narrower confidence intervals and potentially inflated statistical significance of some associations. This limitation has been added to the revised manuscript and should be considered when interpreting the regression findings.

The limitation section was modified as follows: “Additionally, although participants were recruited from ten hospitals, the statistical analysis did not account for potential clustering of patients within hospitals. Patients treated within the same institution may share similar prescribing practices and institutional characteristics, resulting in correlated observations. This may have led to underestimated standard errors, narrower confidence intervals, and potentially inflated statistical significance of some associations; therefore, the regression findings should be interpreted with caution.”

7- The claim that all routes of administration were appropriate requires further support, particularly because 87% of patients received intravenous therapy. Please describe the criteria used to determine route appropriateness and how the ability to tolerate oral or enteral treatment was assessed. Where this information was unavailable, route appropriateness may need to be classified as uncertain rather than appropriate.

Author’s response: Thank you for this important comment. We agree with the reviewer’s concern. The route of administration of ASMs (oral, intravenous, or enteral) was documented during data collection. However, the data collection form did not specifically assess whether patients receiving intravenous therapy were able to tolerate oral or enteral administration or whether a clinical reason necessitating intravenous administration was present. In the original analysis, oral, intravenous, and enteral routes were classified as appropriate routes of administration based on the available guideline recommendations. However, we acknowledge that the appropriateness of the intravenous route cannot be reliably determined without information regarding the patient’s ability to tolerate oral or enteral therapy.

Therefore, after considering the reviewer’s comment, we have removed the assessment of route appropriateness from the analysis rather than classifying routes as appropriate or uncertain retrospectively. The route of administration is now reported descriptively only, without making a judgment regarding its appropriateness. Consequently, route appropriateness has also been removed from the calculation of overall SUP appropriateness. The corresponding Methods, Results, tables, Discussion, and other relevant sections of the manuscript have been revised accordingly.

The revised study limitations are as follows: “Second, although the route of ASM administration was documented, the study did not specifically assess patients’ ability to tolerate oral or enteral therapy or document the clinical reasons necessitating intravenous administration. Therefore, the appropriateness of the route of administration could not be reliably determined and was not included in the appropriateness assessment. Route of administration was consequently reported descriptively only.”

8- The data-availability statement should be strengthened. The manuscript states that no patient identifiers were recorded, but the dataset is available only upon request because of confidentiality and re-identification concerns. Please explain more specifically why a de-identified minimal dataset cannot be shared publicly. If access must remain restricted, objective access conditions and an institutional contact responsible for reviewing requests should be provided.

Author’s response:

Thank you for this important comment. In response to this concern, we consulted the Institutional Review Board (IRB) at Beirut Arab University regarding the public sharing of the study data. We received oral approval from the IRB to share a fully de-identified dataset, provided that no medical record numbers, patient identifiers, or hospital identifiers are disclosed.

Accordingly, we have revised the data availability approach. The dataset underlying the findings of this study has been fully de-identified and is now provided as an Excel file in the Supplementary Appendix. The shared dataset contains no medical record numbers, patient-identifying information, or hospital identifiers, thereby minimizing the risk of re-identification of individual patients or participating institutions.

The Data Availability Statement has been revised accordingly, and the supplementary dataset has been appropriately cited within the manuscript to facilitate transparent access to the data supporting the study findings. As the de-identified dataset is now provided with the manuscript and is no longer available only upon request, restricted-access conditions and an institutional contact for reviewing data requests are no longer applicable.

The Data availability statement reads now: “Data availability statement: All relevant data supporting the findings of this study are available within the manuscript and its Supporting Information files.”

9- Pneumonia and drug interactions are described as adverse effects associated with acid-suppressive therapy. Because this observational study does not appear to include a comparator or formal causality assessment, these events should not be attributed directly to the medications. Please provide the definitions and assessment methods used or describe them more cautiously as events observed during treatment.

Author’s response: Thank you for this important comment. We agree with the you that, given the observational nature of our study and the absence of a comparator group or formal causality assessment, adverse events such as pneumonia and drug-drug interactions cannot be directly attributed to ASMs. Therefore, to avoid implying a causal relationship that cannot be established by our study design, we have removed the assessment and reporting of ASM-associated adverse effects from the manuscript. All corresponding references to these adverse effects have been omitted from the Methods, Results, Discussion, and other relevant sections of the manuscript.

+ Minor comments. Kindly:

- Correct the garbled inappropriate-practice entry in Table 3, which appears to be 402 (77.9%).

Author’s response: Thank you for pointing this out. The garbled entry in Table 3 has been corrected, and the value 402 (77.9%) is now presented appropriately in the revised manuscript.

- Recalculate the route percentages in Table 3; for example, 56 of 516 is approximately 10.9%, not 12%.

Author’s response: Thank you for this important observation. We have recalculated and corrected all route percentages in Table 3 based on the corresponding frequencies and total sample size. The revised percentages have been checked for accuracy and consistency throughout the table.

- Correct the Nephrology entry in Table 4, which presumably should be 1 (0.2%).

Author’s response: Thank you for pointing this out. The Nephrology entry in Table 4 has been corrected to 1 (0.2%) in the revised manuscript.

- Replace the Table 4 footnote referring to “intervention acceptance,” as this is unrelated to the study outcome.

Author’s response: Thank you for this valuable comment. The Table 4 footnote referring to “intervention acceptance” has been replaced with “treatment appropriateness” to accurately reflect the study outcome and ensure consistency with the study objectives.

- Standardize the use of ASM and AST throughout the manuscript.

Author’s response: Thank you for this helpful comment. We have revised the manuscript to standardize the terminology throughout the text. The abbreviation ASM (acid-suppressive medication) is now used consistently throughout the manuscript, and the term AST has been removed.

- Correct the duplicate subsection number “2.7.”

Author’s response: Thank you for highlighting this issue. The duplicate subsection numbering “2.7.” has been corrected, and the subsection numbering has been carefully reviewed for consistency throughout the revised manuscript.

- Reconcile the statement that all eligible patients had an ICU stay with the reported ward distribution and guideline-specific eligibility counts.

Author’s response: Thank you for highlighting this point. We agree that the previous wording stating that all eligible patients had an ICU stay could create confusion when considered alongside the reported ward distribution and the guideline-specific eligibility counts. We have therefore revised this statement and clarified the eligibility results according to each guideline.

The modified statement now reads: “Similarly, according to the ASHP Guidelines 1999, most patients did not have an indication for SUP (n = 426, 77.2%), while 22.8% (n = 126) were considered eligible for SUP, and all eligible patients were critically ill in addition to at least one major or at least two or more minor risk factors for stress ulcer development.”

- Correct typographical and grammatical errors, including “Parentral,” “Data collectors Training,” and the unmatched quotation mark after the SPSS version.

Author’s response: Thank you for highlighting this issue. All the indicated typographical and grammatical errors have been corrected in the revised manuscript, including “Parenteral,” “Data collectors Training,” and the unmatched quotation mark following the SPSS version.

- Consider reporting confidence intervals for the principal prevalence estimates.

Author’s response: Thank you for this valuable suggestion. We have incorporated 95% confidence intervals for the principal prevalence estimates reported in the Discussion. Specifically, 95% confidence intervals were reported for the prevalence estimates from the previously published studies used for comparison, including Lebanon, Ethiopia, Jordan, Sudan, and Saudi Arabia. These estimates are now presented alongside the corresponding percentages in the revised Discussion to provide a clearer representation of the precision of the reported prevalence estimates

The modified section is as follows: “In Lebanon, Zeitoun et al. reported that 67.0% of patients receiving acid-suppressive therapy lacked a SUP indication (95% CI: 64.1–69.9%) (5). Similarly, inappropriate SUP use was reported in 65.7% of patients in Ethiopia (95% CI: 59.3–71.5%) (1), 72.5% in Jordan (95% CI: 65.8–78.3%) (22), and 73.5% in Sudan (95% CI: 68.4–78.1%) (23), suggesting that inappropriate SUP use remains a common challenge across several healthcare settings. Conversely, lower rate was reported in Saudi Arabia, where 46.0% of patients did not meet the criteria for SUP (95% CI: 41.3–50.7%) (24)”.

- Moderate statements about increased costs and adverse effects, as these outcomes were not directly evaluated in this study.

Author’s response: Thank you for this important comment. We have revised the manuscript to moderate the statements regarding potential adverse effects and increased healthcare costs. The Discussion and Conclusion now focus on the inappropriate prescribing and continuation of ASMs identified in our study without making unsupported claims regarding adverse effects or healthcare costs.

Overall, this study has the potential to provide useful information on stress ulcer prophylaxis practices in Lebanon. Addressing the inconsistencies in the cohort definition, guideline classification, duration assessment, and statistical analysis would substantially improve the reliability and clarity of the manuscript.

Author’s response: Thank you for your positive assessment and constructive feedback. We have carefully addressed the concerns regarding cohort definition, guideline classification, duration assessment, and statistical analysis. The relevant sections of the manuscript have been revised accordingly to improve the consistency, methodological rigor, clarity, and reliability of the study findings. We sincerely appreciate your valuable comments, which have helped strengthen the manuscript.

________________________________________

6. PLOS authors have the option to publish the peer review history of their article (what does this mean?). If published, this will include your full peer review and any attached files.

If you choose “no”, your identity will remain anonymous but your review may still be made public.

Do you want your identity to be public for this peer review? For information about this choice, including consent withdrawal, please see our Privacy Policy.

Reviewer #1: No

Reviewer #2: No

Attachment Submitted filename: Responses to reviewers comments SUP R.docx

# Editor report: Decision Letter 1

**DOI:** 10.1371/journal.pone.0361969.r003

**Authors:** Jad El Masri

19 Oct 2026

<p>Assessment of the Appropriateness of Stress Ulcer Prophylaxis Practice among Hospitalized Patients across Lebanese Hospitals: A Multicenter Prospective Study

PONE-D-26-27216R1

Dear Dr. Itani,

We’re pleased to inform you that your manuscript has been judged scientifically suitable for publication and will be formally accepted for publication once it meets all outstanding technical requirements.

Within one week, you’ll receive an e-mail detailing the required amendments. When these have been addressed, you’ll receive a formal acceptance letter and your manuscript will be scheduled for publication.

An invoice will be generated when your article is formally accepted. Please note, if your institution has a publishing partnership with PLOS and your article meets the relevant criteria, all or part of your publication costs will be covered. Please make sure your user information is up-to-date by logging into Editorial Manager at Editorial Manager® and clicking the ‘Update My Information' link at the top of the page. For questions related to billing, please contact billing support.

If your institution or institutions have a press office, please notify them about your upcoming paper to help maximize its impact. If they’ll be preparing press materials, please inform our press team as soon as possible -- no later than 48 hours after receiving the formal acceptance. Your manuscript will remain under strict press embargo until 2 pm Eastern Time on the date of publication. For more information, please contact onepress@plos.org.

Kind regards,

Jad El Masri

Academic Editor

PLOS One

Additional Editor Comments (optional):

Reviewers' comments:

# Editor report: Acceptance letter

**DOI:** 10.1371/journal.pone.0361969.r004

**Authors:** Jad El Masri

PONE-D-26-27216R1

PLOS One

Dear Dr. Itani,

I'm pleased to inform you that your manuscript has been deemed suitable for publication in PLOS One. Congratulations! Your manuscript is now being handed over to our production team.

At this stage, our production department will prepare your paper for publication. This includes ensuring the following:

* All references, tables, and figures are properly cited

* All relevant supporting information is included in the manuscript submission,

* There are no issues that prevent the paper from being properly typeset

You will receive further instructions from the production team, including instructions on how to review your proof when it is ready. Please keep in mind that we are working through a large volume of accepted articles, so please give us a few days to review your paper and let you know the next and final steps.

Lastly, if your institution or institutions have a press office, please let them know about your upcoming paper now to help maximize its impact. If they'll be preparing press materials, please inform our press team within the next 48 hours. Your manuscript will remain under strict press embargo until 2 pm Eastern Time on the date of publication. For more information, please contact onepress@plos.org.

You will receive an invoice from PLOS for your publication fee after your manuscript has reached the completed accept phase. If you receive an email requesting payment before acceptance or for any other service, this may be a phishing scheme. Learn how to identify phishing emails and protect your accounts at https://explore.plos.org/phishing.

If we can help with anything else, please email us at customercare@plos.org.

Thank you for submitting your work to PLOS One and supporting open access.

Kind regards,

PLOS One Editorial Office Staff

on behalf of

Dr. Jad El Masri

Academic Editor

PLOS One
